# Integrating *In Vitro* Data and Physiologically Based Kinetic Modeling to Predict and Compare Acute Neurotoxic Doses of Saxitoxin in Rats, Mice, and Humans

**DOI:** 10.1021/acs.est.3c01987

**Published:** 2023-07-21

**Authors:** Jiaqi Chen, Annelies Noorlander, Sebastiaan Wesseling, Hans Bouwmeester, Nynke I. Kramer, Ivonne M.C.M. Rietjens

**Affiliations:** Division of Toxicology, Wageningen University and Research, Stippeneng 4, Wageningen, Gelderland 6708 WE, The Netherlands

**Keywords:** saxitoxin, neurotoxicity, physiologically based kinetic (PBK) model, quantitative *in vitro* to *in vivo* extrapolation (QIVIVE), risk assessment

## Abstract

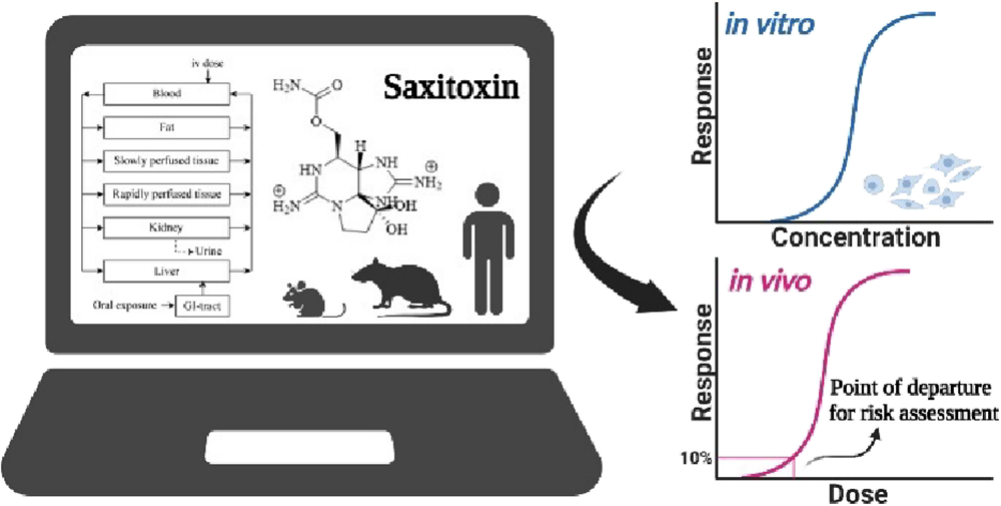

Current climate trends are likely to expand the geographic distribution of the toxigenic microalgae and concomitant phycotoxins, making intoxications by such toxins a global phenomenon. Among various phycotoxins, saxitoxin (STX) acts as a neurotoxin that might cause severe neurological symptoms in mammals following consumptions of contaminated seafood. To derive a point of departure (POD) for human health risk assessment upon acute neurotoxicity induced by oral STX exposure, a physiologically based kinetic (PBK) modeling-facilitated quantitative *in vitro* to *in vivo* extrapolation (QIVIVE) approach was employed. The PBK models for rats, mice, and humans were built using parameters from the literature, *in vitro* experiments, and *in silico* predictions. Available *in vitro* toxicity data for STX were converted to *in vivo* dose–response curves via the PBK models established for these three species, and POD values were derived from the predicted curves and compared to reported *in vivo* toxicity data. Interspecies differences in acute STX toxicity between rodents and humans were found, and they appeared to be mainly due to differences in toxicokinetics. The described approach resulted in adequate predictions for acute oral STX exposure, indicating that new approach methodologies, when appropriately integrated, can be used in a 3R-based chemical risk assessment paradigm.

## Abstract 2

An integration of PBK modeling-facilitated QIVIVE and *in vitro* toxicity data is a promising alternative to animal testing for human health risk assessment upon exposure to phycotoxins.

## 1 Introduction

**Figure 1:**
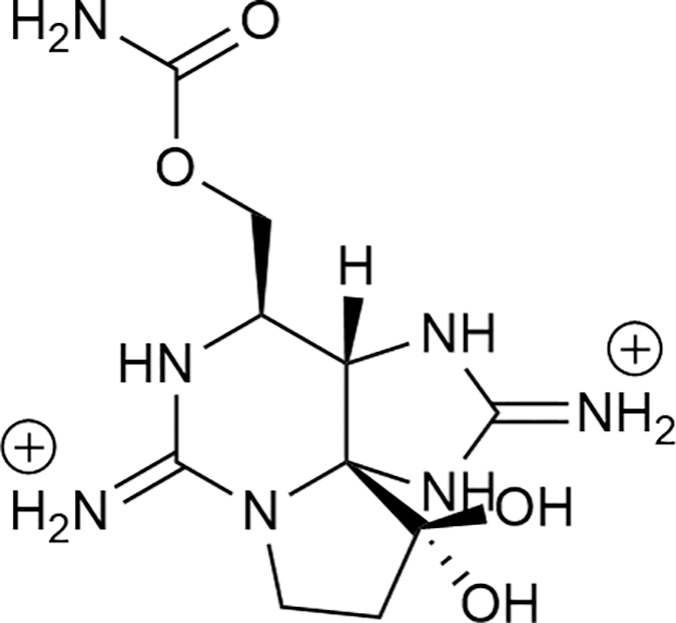
Chemical structure of saxitoxin (STX).

Saxitoxin (STX) is a naturally occurring tetrahydropurine phycotoxin (Figure 1) produced mainly by marine dinoflagellates and some freshwater cyanobacteria, acting as a selective blocker to voltage-gated sodium channels on excitable membranes.^1,2^ Nearly 60 structural analogues of STX have been found so far, which together are referred to as STX-group toxins.^3^ Bivalve mollusks ingest STX-producing organisms via filter-feeding, which results in STX bioaccumulation and transfer through the food web, potentially causing neurotoxicity in marine wildlife and humans.^4^ Documented human poisonings have notably increased since 1941.^5^ More than 400 human poisoning cases have been reported between 2001 and 2015,^6^ and the reports increased by more than 3.5 times in the decade 2011–2020 compared to previous periods.^5^

Upon oral administration to mammals, STX will be absorbed from the gastrointestinal tract and rapidly distributed to body fluids and tissues.^3,7^ Information about STX metabolism is limited. Oxidation and glucuronidation of STX, albeit slow, were reported in an *in vitro* human liver microsome incubation study.^8^ Urinary excretion of unchanged STX is identified as the major excretion route.^9−11^ After acute exposure to STX, neurological and muscular symptoms will appear within minutes to hours, including a prickly sensation in limbs, headache, nausea, muscular paralysis, and sometimes even death due to respiratory paralysis.^3,7^ Most symptoms are believed to be attributed to peripheral effects, while the toxic effects of STX on the central nervous system have yet to be confirmed.^3,7^

Based on the acute toxic potency compared with STX following intraperitoneal injection in mice, a specific toxicity equivalency factor (TEF) was set for each STX analogue, so that their combined exposure and total toxicity can be calculated as the sum value expressed in STX equivalents given that their affinities to voltage-gated sodium channels differ.^1−3^ Currently, the acute reference dose (ARfD) for STX-group toxins is established based on data from human cases because acute toxicity studies in rats or mice mainly focus on the median lethal dose for 50% of the population (LD_50_) and no dose–effect relationships are provided.^12^ ARfDs of 0.5 and 0.7 μg STX equivalents/kg BW have been established by EFSA^3^ and the joint FAO/IOC/WHO ad hoc Expert Consultation,^13^ respectively, based on the lowest-observed-adverse-effect levels (LOAELs) of 1.5 and 2 μg STX equivalents/kg BW for mild toxic symptoms. An uncertainty factor of 3 was used for the conversion of the LOAELs to no-observed-adverse-effect levels (NOAELs). No additional uncertainty factors were deemed necessary for interindividual variation given that the point of departure (POD) was derived from human intoxication cases covering a large number of affected consumers including the most sensitive individuals.^3,13^

Thus, the actual database based on which the ARfDs have been established appears rather limited, and it is of interest to investigate whether new approach methodologies (NAMs) could be of use to further support these health-based guidance values. Physiologically based kinetic (PBK) modeling-facilitated quantitative *in vitro* to *in vivo* extrapolation (QIVIVE) has been increasingly applied as an alternative to animal testing in accordance with the principles of Next Generation Risk Assessment.^14^ The approach provides a sound scientific basis to extrapolate across species, routes of exposure, and exposure scenarios^15,16^ and has been employed for predicting *in vivo* toxicity of chemicals with various endpoints, contributing to their hazard characterization and risk assessment.^15−19^ With this approach, data from *in vitro* concentration-based toxicity curves can be translated to *in vivo* dose–response curves, from which a POD for risk assessment can be derived.^15,16^ In the present work, this approach, integrated with available *in vitro* toxicity data for STX derived from rat, mouse, and human cell models, was employed to investigate whether a POD could be derived for the risk assessment upon acute STX exposure. The results obtained will provide insights into the quality of the QIVIVE-based predictions and into potential interspecies differences in acute STX toxicity between rodents and humans.

## 2 Materials and Methods

### 2.1 Rat Hepatocyte Incubation Assay

Primary rat hepatocytes were incubated with STX to investigate the *in vitro* intrinsic clearance of STX, and the remaining STX after incubation was quantified by LC–MS/MS (Supporting Information).

### 2.2 Procedure for POD Derivation

The approach used in the present study to derive a POD for risk assessment upon acute STX exposure consists of the following steps:^15,16^ (1) establishing rat, mouse, and human PBK models for STX; (2) evaluating model performance by comparing model predictions to available toxicokinetic data in rats, mice, and humans; (3) collecting concentration-based STX toxicity data from *in vitro* cell models of these three species; (4) correcting for differences in protein binding of STX in the *in vitro* assays and the *in vivo* situations; (5) setting the effective *in vitro* STX concentrations equal to the unbound maximum STX concentrations in the target tissue and calculating corresponding dose levels by PBK modeling-based reverse dosimetry; (6) assuming the toxic effect at the *in vitro* concentration and the corresponding *in vivo* dose to be similar, thereby enabling definition of the *in vivo* dose–response curve; and (7) performing benchmark dose (BMD) analysis on the predicted dose–response curve to derive a POD and comparing the POD to available *in vivo* toxicity data.

### 2.3 PBK Model Development

**Figure 2:**
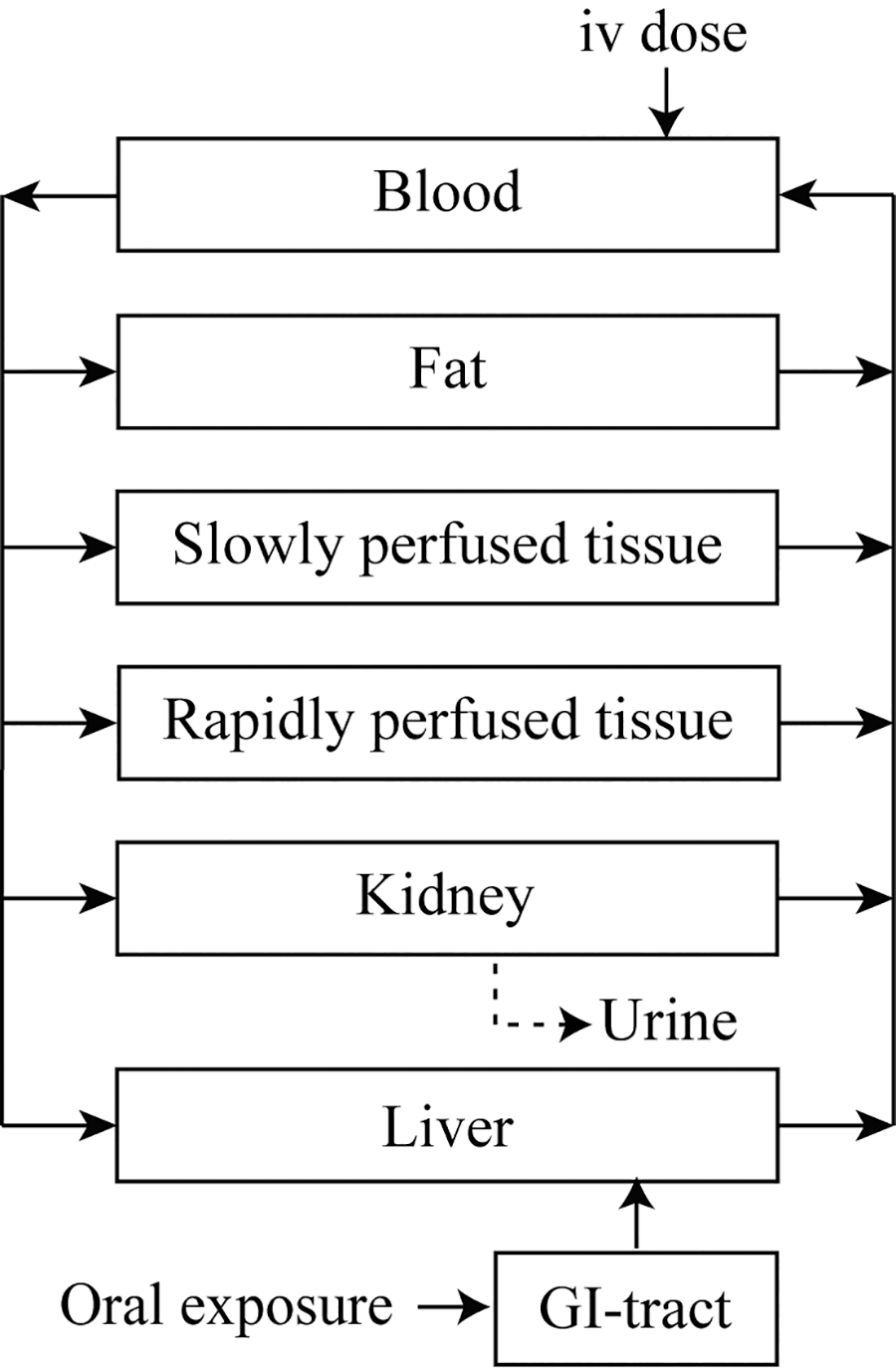
Schematic representation of the PBK model for STX.

PBK models describing the toxicokinetics of STX for rodents and humans were developed based on a model for tetrodotoxin (TTX) with some modifications.^20^ As shown in the conceptual model presented in Figure 2, the PBK model contained liver, blood, kidney, fat, slowly perfused tissue, and rapidly perfused tissue compartments. Species-specific physiological parameters are summarized in Table S1. Considering that the toxic effects following acute STX exposure are mostly reported in the peripheral nervous system^3,7^ and that STX can readily pass the blood–brain barrier,^10^ the brain was combined into the rapidly perfused tissue compartment in our models instead of being listed as an independent compartment, and the STX concentration in blood was used for further QIVIVE. The oral route was included in the model considering that human exposure to STX is usually through contaminated seafood, and the intravenous (iv) route was added to enable model evaluation by comparison to experimental time-urinary excretion profiles in rats upon iv dosing.^9,21^ Though the conceptual models for rats, mice, and humans are similar, parameters that acknowledge species-related toxicokinetics were defined when establishing species-specific PBK models as a well-accepted approach for studying interspecies differences with PBK modeling.^15,16^

Information about the oral bioavailability and absorption rate of STX in rodents and humans is limited. Gonyautoxin 2/3 epimers (GTX2/3), representing carbamoyl analogues of STX, were reported to passively permeate human intestines.^22^ Based on this information, STX is assumed to diffuse passively across the intestinal tissue. An approach based on quantitative structure–activity relationships (QSARs) was applied for the calculation of the uptake rate constant (ka, in h^–1^) and the fraction absorbed (Fa) for STX,^23,24^ given that no relevant information is available. Briefly, a QSAR-predicted *in vitro* apparent permeability constant (LogP_app_) for Caco-2 cells (−0.241 × 10^–6^ cm/s)^25^ was scaled to ka and Fa based on the following equations (eqs 1–4):
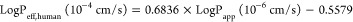
1
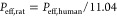
2
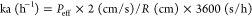
3
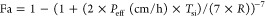
4where eq 1 describes the *in vitro* to *in vivo* scaling of the Caco-2 apparent permeability (LogP_app_) to a human effective permeability (LogP_eff_) for passively diffusive compounds.^26^ The rat *P*_eff_ was defined using eq 2 with the human *P*_eff_ and an interspecies scaling factor.^23^ With eqs 3 and 4,^27^ along with an *R* (the radius of the small intestine) of 0.18 cm for rats or 1 cm for humans,^23,24^ and a *T*_si_ (the small intestinal transit time) of 1.47 h for rats or 3.32 h for humans,^23,24^ the *P*_eff_ value was converted to a ka and an Fa, which were 0.07 h^–1^ and 0.1 for rats and 0.14 h^–1^ and 0.36 for humans, respectively. The calculated Fa thus obtained for STX in rats appeared comparable to the reported oral bioavailability (0.067) of the related compound TTX in a study, where pellets containing TTX were fed to rats.^28^ For mice, the ka and Fa were set equal to the rat parameters.

To describe STX distribution to the tissues, the tissue:blood partition coefficients (*P*) were required in the model (Table S1), which were predicted with the QIVIVE tool (version 2.0)^29^ based on the Rodgers and Rowland method and input parameters including lipophilicity (LogP), acid–base properties (p*K*_a_), and the fraction unbound in plasma (fup). The LogP value of STX is −4.6,^7^ pKa values are 8.22 and 11.28,^30^ and the fup value is 0.9 predicted by an online tool.^31^ The blood plasma ratio (BPr) is assumed to be 1 for basic compounds, like STX in the current study, when experimental data are not available.^32^ Thus, no correction was needed when comparing blood and plasma STX concentrations, and the predicted tissue:plasma partition coefficients in rats and humans obtained from the QIVIVE tool were used directly in the PBK model (Table S1). The partition coefficients for the mouse model were assumed to be the same as those for the rat model.

Renal clearance was used for describing the elimination of STX from the systemic circulation since the hepatic clearance of STX was found to be limited (see Section 3.1). The renal excretion defined as the glomerular filtration rate times the venous blood concentration of STX leaving the kidney compartment was used in rat, mouse, and human models, as this process was reported as a primary way for STX clearance.^10^ The glomerular filtration rates of rats, mice, and humans used in the models were 5.2, 14, and 1.8 mL/min/kg BW, respectively.^33^

All three models were coded and numerically integrated in Berkeley Madonna (version 10.2.8, UC Berkeley, CA, USA), applying Rosenbrock’s algorithms for stiff systems (Supporting Information).

### 2.4 PBK Model Evaluation

*In vivo* blood and urine concentration–time profiles of STX were collected from the literature and used to evaluate the model performance. For the rat model evaluation, two reported datasets of time-dependent cumulative urinary excretion of STX after iv administration were available for comparison with the model simulations.^9,21^ For the human model, blood concentrations and urinary concentrations from poisoning cases were available for model evaluation.^11,34,35^ No mouse data were available for the mouse model evaluation. TechDig 2.0 was used to extract and convert graphic data from the reported *in vivo* and *in vitro* studies to a numerical format.

A local sensitivity analysis was performed to identify the influential parameters on the predicted maximum blood STX concentration as a model output for QIVIVE (Supporting Information).

### 2.5 Model Application: QIVIVE

*In vitro* concentration-dependent toxicity data for STX from rat, mouse, and human cell models were collected (Tables S2–S4). STX is a known blocker to voltage-gated sodium channels and is reported to cause most toxic effects in peripheral systems;^3,7^ however, data from all available *in vitro* toxicity assays for STX in rodent cell models, including cell cultures from brain tissues, were used for the QIVIVE. The *in vitro* models included rat neonatal cortical cultures,^36^ rat voltage-gated sodium channels (i.e., rNav1.4) expressed in stably transfected CHO or HEK-293 cells,^37^ mouse frontal cortex cultures,^38^ mouse cerebellar granule cell cultures,^39^ mouse brain synaptoneurosomes,^40^ and human voltage-gated sodium channels (i.e., hNav1.6) expressed in stably transfected CHO or HEK-293 cells.^2^ In all these studies, electrophysiological signal changes under increasing STX concentrations were recorded using the multielectrode (MEA) assay and patch-clamp technique or monitored with a voltage-sensitive fluorescent probe. Additionally, *in vitro* toxicity data were also obtained from a binding assay characterizing the competitive binding of STX against tritium-labeled STX for receptor sites on rat brain synaptosomes^41^ or from the cytotoxicity assay in mouse Neuro-2a cells in the presence or absence of ouabain and veratridine as a sodium/potassium ATPase pump blocker and a sodium channel activator, respectively.^42^

To these available *in vitro* concentration–response curves (Tables S2–S4), PBK modeling-facilitated QIVIVE was applied to translate *in vitro* concentration–response relationships to rat, mouse, and human bioequivalent dose–response relationships. The *in vitro* unbound concentration of STX tested in the reported *in vitro* assays was set equal to the unbound maximum concentration of STX in blood as indicated in eq 5:
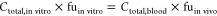
5where *C*_total, *in vitro*_ is the STX concentration tested *in vitro* and the *in vitro* unbound fraction (fu_*in vitro*_) was assumed to be 1 given the negligible protein levels used in the media for *in vitro* toxicity assays; *C*_total, blood_ is the maximum total STX concentration in blood, and the fu_*in vivo*_ was set equal to the predicted fup (0.9). Reverse dosimetry on each effective concentration (*C*_total, blood_) was performed using the established PBK models to derive the corresponding oral STX dose level, enabling the translation of the *in vitro* concentration-dependent data to an *in vivo* dose–response curve.^15,23,24^

### 2.6 BMD Analysis

To evaluate the model performance, PBK modeling-based predictions for acute STX toxicity were compared with available *in vivo* toxicity data in both rodents and humans. To this end, BMD modeling was used to derive PODs from the predicted *in vivo* dose–response curves. The Benchmark Dose Software (BMDS, version 3.2) developed by the US EPA was employed for the BMD analysis with a hill or exponential model, a benchmark response (BMR) for extra risk, a confidence level of 0.95, a distribution type of normal, and a variance type constant. The value resulting in a 10% BMR change with a 95% lower confidence limit (BMDL_10_) from the model that provided the best fit (lowest AIC) to the data was chosen and was further evaluated against reported NOAELs. Likewise, the value resulting in a 50% BMR change (BMD_50_) was also derived and compared with available LD_50_ values.

## 3 Results

### 3.1 Hepatic Clearance of STX

To study the hepatic clearance of STX, rat hepatocytes in suspension were incubated with 1 μM STX, a nontoxic concentration to the hepatocytes (102.7 ± 4.0% viability compared to the control after a 2 h exposure), and the results obtained reveal that the metabolism was negligible (Figure S1). For humans, the glucuronidation of STX was reported in an *in vitro* incubation study with human liver microsomes and was regarded as a detoxification pathway for STX excretion.^8^ The catalytic efficiency of this pathway, calculated as the corresponding *V*_max_ (9.7 ± 2.8 × 10^–3^ pmol/min/mg microsomal protein) divided by the *K*_m_ (32.02 ± 0.64 μM), was 0.30 × 10^–3^ μL/min/mg microsomal protein,^8^ suggesting that STX was also metabolized to only a limited extent in the human liver. Given that no substantial turnover of STX was observed in both rat hepatocyte and human liver microsome assays, the hepatic clearance of STX was considered to be negligible relative to renal clearance. As no data on mouse hepatic clearance were available, a similar assumption was made for mice. Therefore, only renal clearance of STX was taken into account when developing the rat, mouse, and human PBK models.

### 3.2 PBK Model Evaluation

**Figure 3:**
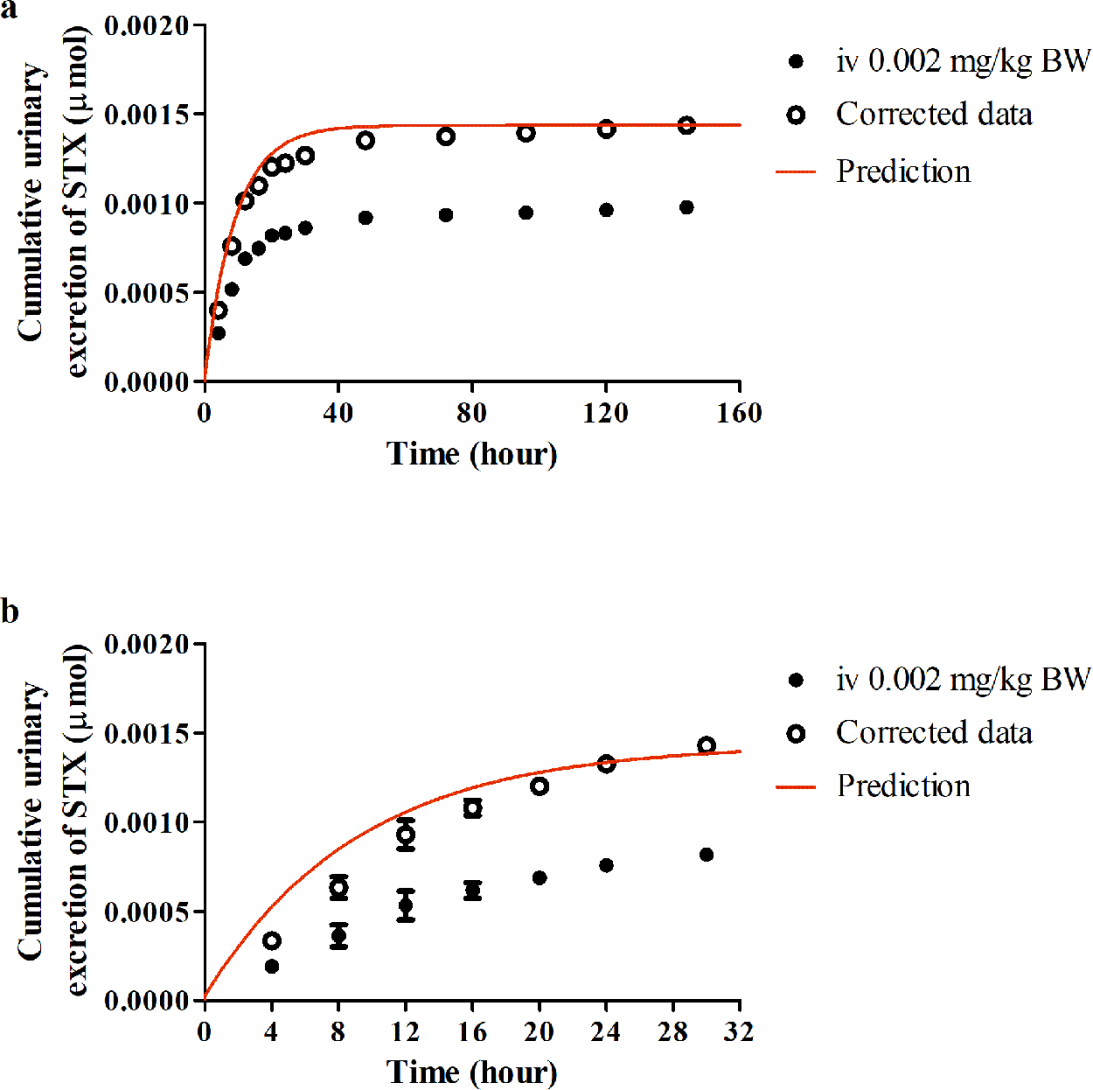
Comparisons between PBK model predictions and reported *in vivo* data for time-dependent cumulative urinary STX excretion in rats during (a) 144 and (b) 30 h following iv administration of STX at 0.002 mg/kg BW.^9,21^ Reported data (closed symbols) and data after correcting for STX recovery of the dose administered (open symbols) are also presented. Data points represent means ± SEM (where available).

The established PBK models were first evaluated by comparing model predictions with available *in vivo* kinetic data to ensure their good performance before being used for QIVIVE. Two datasets of time-dependent cumulative urinary excretion of STX upon iv administration in rats (0.002 mg/kg BW) were available for the evaluation of the rat model.^9,21^ The model predictions are within 2-fold from the reported urinary STX excretion in the 144 h following administration (Figure 3a). Interestingly, the total excreted STX in this study was reported as approximately 68% of the total administered dose for the full study period, probably due to the low recovery rate of the analytical method used.^9^ After correction of the reported *in vivo* data to full recovery of the administered dose by the analytical method, a result to be expected upon iv dosing, the differences between the predicted and observed urinary STX excretion decrease to 1.3-fold (Figure 3a). Similarly, the differences between the model-predicted urinary STX excretion during 30 h after administration and the *in vivo* data from another study with an iv dosing of 0.002 mg STX/kg BW in rats^21^ are within 2.7-fold and decrease to 1.6-fold when compared to the *in vivo* recovery-corrected data (Figure 3b). These results suggest that the rat PBK model with only glomerular filtration as a clearance process of STX could well-simulate the urinary STX excretion.

**Figure 4:**
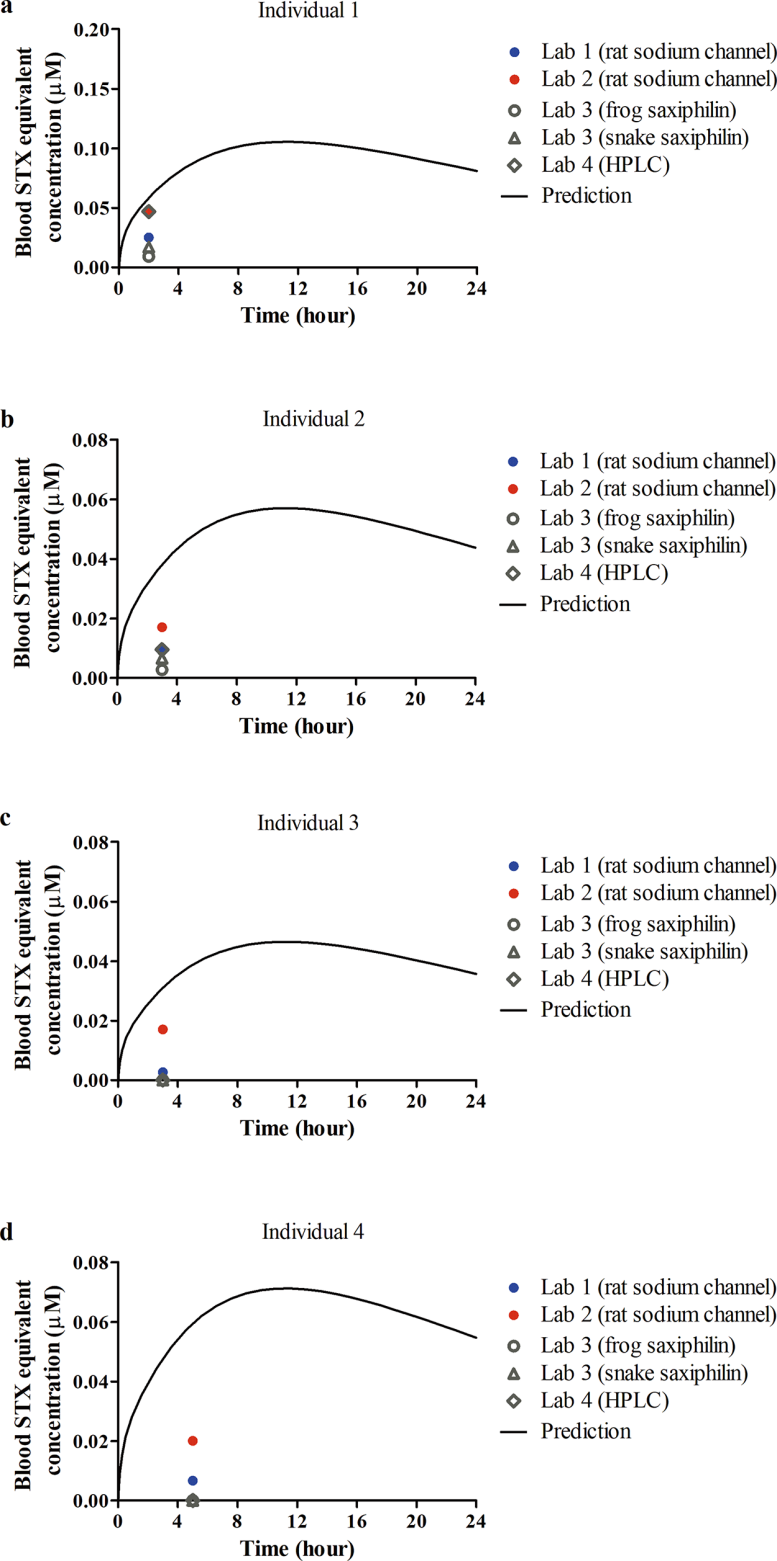
Comparisons between PBK modeling-based predictions and reported *in vivo* data for time-dependent blood STX equivalent concentrations in humans after 2, 3, 3, and 5 h upon acute oral exposure at (a) 0.34, (b) 0.184, (c) 0.15, and (d) 0.23 mg STX equivalents/kg BW, respectively.^11^ Results from different labs are presented in different colors or symbols, and the curves present PBK modeling-based predictions at the relevant dose levels.

The human PBK model was built based on the rat model and was evaluated by comparison of predicted blood levels of STX against a set of data on the time-dependent blood STX concentrations collected from poisoning cases, where humans might have been coexposed to STX and other naturally existing STX analogues.^11^ In this study, the total concentrations of STX-group toxins in the contaminated mussels were determined using the standard mouse bioassay, and the estimated exposure dose levels for four individuals were expressed in STX equivalents, amounting to 0.15, 0.184, 0.23, and 0.34 mg STX equivalents/kg BW.^11^ The reported blood concentrations expressed in STX equivalents in samples from the four individuals were determined using four different methods at four independent laboratories, and the results showed substantial variations (Figure 4). The results using HPLC analysis at laboratory 4 were reported by the study authors to be questionable, and the toxin levels reported by laboratory 1 and 2 were considered by the authors to be in line with the severity of illness of the four individuals. The model predictions are close to the results from laboratory 2, where the predicted values are 1.2-, 2.2-, 1.8-, and 3.0-fold higher compared to the reported data (Figure 4), which are acceptable given the potential uncertainties from dose estimation and analysis methods.^3^

The human PBK model predictions were further evaluated by comparison of predictions to results from a study reporting urinary STX concentrations in humans, where urine samples from two individuals in a poisoning case were collected within 4 h after shellfish consumption.^34^ The total concentrations of STX-group toxins in the contaminated mussels were determined with the mouse bioassay and expressed in STX equivalents, while only STX was quantified in the urine samples. The dose levels were estimated as 0.1007 and 0.2854 mg STX equivalents/kg BW for the two individuals,^43^ and the urinary STX concentrations were reported as 0.39 and 0.05 μM, respectively,^34,35^ which were unexpected as the higher dose level resulted in a lower urinary STX level. To allow comparison of the reported data to the model predictions, the available urinary concentrations were converted to the amount of STX excreted within 4 h using a normal urine production of 83 mL/h^44^ with an assumption that no urination occurred before the sample collection, resulting in 0.13 and 0.017 μmol of STX in urine. The model-predicted urinary STX excretion within the first 4 h amounted to 0.37 and 1.06 μmol at the estimated dose levels of 0.1007 and 0.2854 mg STX equivalents/kg BW, respectively, values that were 2.8- and 62.4-fold higher than the amounts reported to be excreted by the two individuals. Thus, it appears that the PBK model seemed to overpredict the human urinary excretion, especially that of the individual who ingested an estimated dose level of 0.2854 mg STX equivalents/kg BW but showed milder symptoms and an 8-fold lower urinary STX concentration than the individual, despite an estimated 2.8-fold lower dose level (0.1007 mg STX equivalents/kg BW). These deviations between the two case studies and also between the reported and PBK model-predicted levels might be ascribed to interindividual differences, the high degree of uncertainty in the actual dose levels, potential urination before sampling, and to the fact that the dose levels expressed in STX equivalents also included STX analogues, while in the urinary samples, only STX was quantified.

Taking all comparisons into consideration, it was concluded that the rat and human PBK models were able to provide adequate predictions of time-dependent blood and urinary concentration profiles. The mouse model established based on the rat model, but with the mouse physiological parameters, was assumed to perform well, though no *in vivo* mouse kinetic data were available for this evaluation. Following a local sensitivity analysis (Figure S2), the models for these three species were subsequently used for QIVIVE and further evaluated by comparison of obtained predictions to reported *in vivo* toxicity data.

### 3.3 Evaluation of Predicted *In Vivo* Dose-Dependent Response and PODs

**Figure 5:**
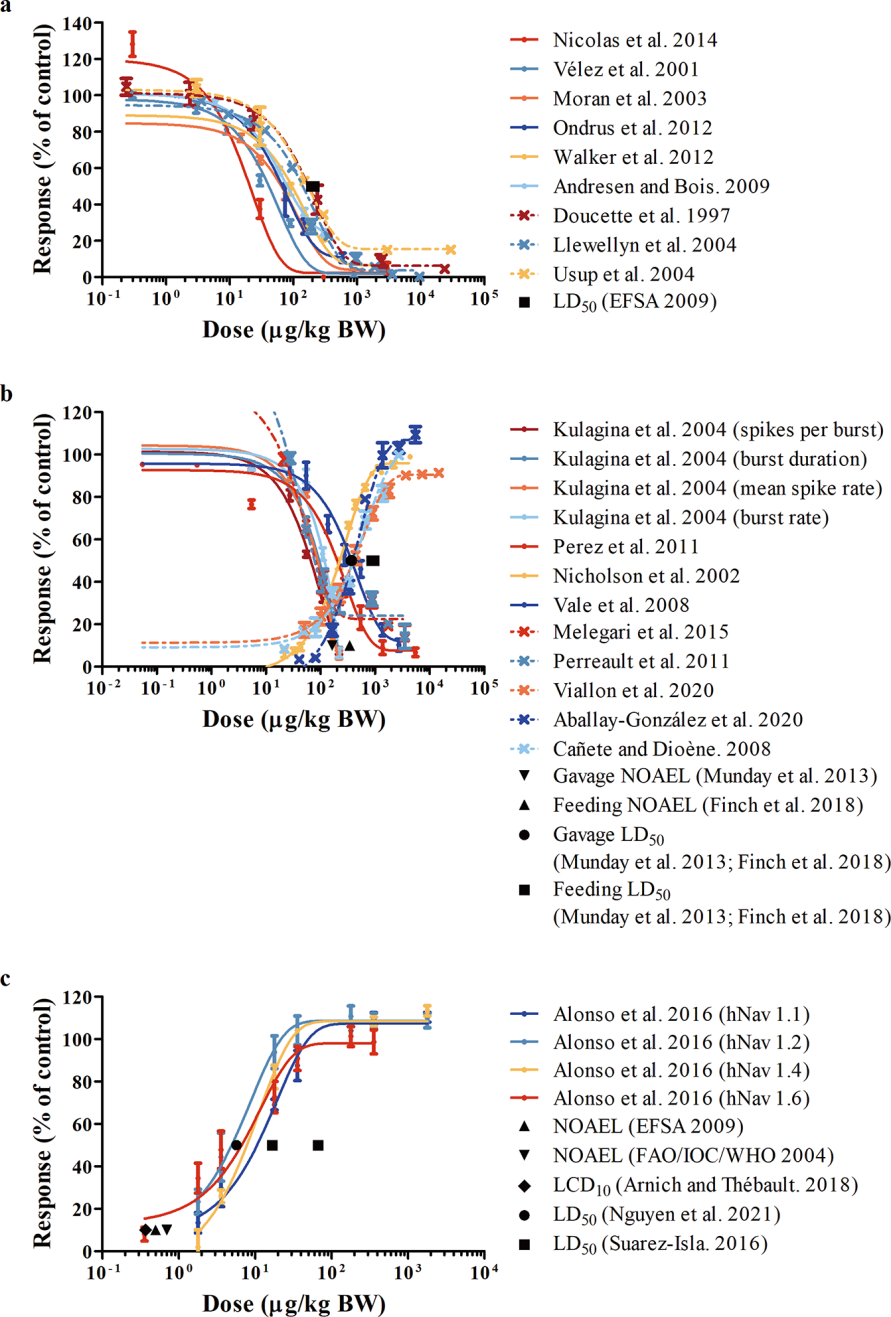
Reported data for comparison (NOAEL, LD_50_, and LCD_10_) and color-coded predicted *in vivo* dose–response curves obtained by PBK modeling-facilitated QIVIVE of the *in vitro* concentration–response curves taken from the respective references for (a) rats, (b) mice, and (c) humans. Details are summarized in Tables S2–S5.

Available *in vitro* toxicity data for rats, mice, and humans (Tables S2–S4) were converted to *in vivo* dose-dependent curves using PBK modeling-facilitated QIVIVE (Figure 5). Given that the sensitivity of the seven individual human sodium ion channel subtypes used in the *in vitro* assay for STX toxicity evaluation varied up to 408-fold according to the half-maximal inhibitory concentrations (IC_50_),^2^ only datasets from the four subtypes (hNav1.1, 1.2, 1.4, and 1.6) that showed high sensitivity to STX exposure were used for the QIVIVE and the subsequent BMD analysis for humans. Reported LD_50_ values and also NOAELs based on the occurrence of toxic symptoms as an endpoint were collected for comparison (Figure 5 and Table S5). No rat NOAEL is available, and the rat LD_50_ is reported as 192–212 μg/kg BW.^3^ Mouse NOAELs are 163 and 341 μg/kg BW upon administration of STX via gavage and feeding, respectively, and LD_50_ values upon gavage dosing are 356 and 370 μg/kg BW, while upon feeding, the LD_50_ values are 853 and 958 μg/kg BW.^45,46^ For humans, reported NOAELs are 0.5 and 0.7 μg STX equivalents/kg BW.^3,13^ In addition, Arnich and Thébault^43^ collected epidemiological data from previous intoxication cases associated with the ingestion of shellfish contaminated with STX-group toxins and built a dose–symptom model, resulting in identification of a dose of 0.37 μg STX equivalents/kg BW as the lower critical dose with a probability higher than 10% of showing symptoms (LCD_10_). The human LD_50_ was reported as 5.7 μg/kg BW by Nguyen et al.^47^ or estimated by Suarez-Isla^48^ as 1–4 mg depending upon age and physical conditions of the individual, amounting to 16.7–66.7 μg/kg BW assuming a body weight of 60 kg for the average individual. All these data are also presented in Figure 5 for comparison to the predicted *in vivo* dose–response curves, which shows that the predicted curves derived from available *in vitro* concentration–response datasets are in line with these PODs.

**Figure 6:**
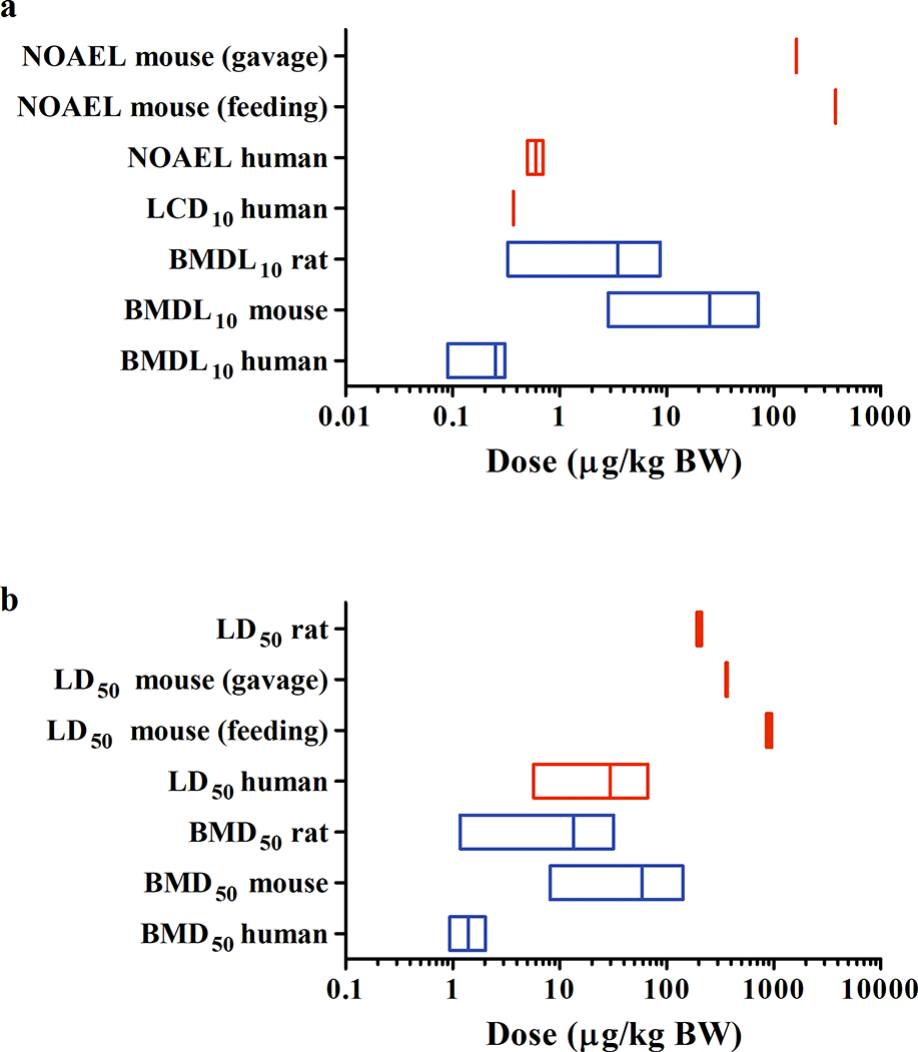
Comparison of the reported (red) and predicted (blue) points for comparison upon acute oral exposure to STX in rats, mice, and humans (details are summarized in Tables S2–S5) based on (a) NOAELs, LCD_10_, and BMDL_10_ values representing the reported no-observed-adverse-effect levels, the reported lower critical dose causing 10% response, and the predicted benchmark 95% lower confidence limit of the dose levels causing 10% response obtained by PBK modeling-facilitated QIVIVE, respectively, and (b) LD_50_ and BMD_50_ values representing the reported median lethal dose and the predicted benchmark dose level causing 50% response by PBK modeling-facilitated QIVIVE, respectively. The *y*-axis represents the legend for the corresponding box plot.

To further evaluate model predictions and derive PODs for risk assessment upon acute oral exposure to STX, BMDL_10_ and BMD_50_ values for rats, mice, and humans were derived from the predicted dose–response curves (Tables S2–S4), and the reported and predicted data for these three species are presented in Figure 6. The results suggest that rats and mice show comparable sensitivity toward acute oral STX exposure, while lower NOAEL and LD_50_ values were reported for humans than for rodents, indicating that humans are more susceptible to the acute toxicity of STX as corroborated by the predictions. The predicted PODs for rodents are lower than reported NOAELs and LD_50_ values, suggesting that the *in vitro* rodent models responded sensitively toward STX exposure. The average prediction of the BMDL_10_ amounts to 25.4 μg/kg BW for mice and 3.5 μg/kg BW for rats (Tables S2 and S3). The obtained mean BMDL_10_ for humans is 0.25 μg/kg BW (Table S4), which is 2-, 2.8-, and 1.5-fold lower compared to the NOAELs derived by EFSA,^3^ the joint FAO/IOC/WHO ad hoc Expert Consultation,^13^ and the LCD_10_ reported by Arnich and Thébault,^43^ respectively. The average predicted human BMD_50_ obtained is 1.4 μg/kg BW (Table S4), which is 4.1-fold lower than the lowest human LD_50_ reported by Nguyen et al.^47^

## 4 Discussion

In the present study, we applied the PBK modeling-facilitated QIVIVE approach for predicting *in vivo* acute neurotoxicity induced by a phycotoxin (STX) in rodents and humans and for deriving a POD for its risk assessment to further support the current ARfDs.^3,13^ To this end, PBK models were first developed for rats, mice, and humans, and the validated models were then used for converting concentration–response datasets obtained from *in vitro* cell models to corresponding *in vivo* dose-STX toxicity curves, from which PODs were derived via BMD analysis and compared to reported NOAEL and LD_50_ values.

STX is assumed to be absorbed via passive diffusion by the intestine in analogy to what was reported for its structural analogues.^22^ The fraction of dose absorbed (Fa) and uptake rate constant (ka) of STX needed for establishing the human PBK model were obtained from *in silico* methods based on the *in vivo*/*in vitro* drug permeability correlation between the human jejunum (*in vivo*) and Caco-2 cells (*in vitro*).^25,26^ This implies that the intestinal absorption parameters for humans could be derived from an *in vitro* determined or a QSAR-predicted Caco-2 apparent permeability constant.^23−25^ Corresponding rodent parameters were scaled from human values by taking into account the species-specific radius and transit time of the small intestine.^23,24^ This *in silico* approach is promising in predicting intestinal absorption parameters for chemicals, but care should still be taken given that the correlation might be poor especially for carrier-mediated chemicals, as significant differences in gene expression levels of several transporters (i.e., organic cation transporters) were observed between Caco-2 cells and human intestines.^26^ Except for intestinal absorption, absorption of STX-group toxins through mucous membranes of the mouth was proposed to occur, based on the observation that the local numbness appeared within minutes after eating toxic food;^3,7^ however, this local absorption was not included in our models due to limited information on its relative importance and related rate constants. Interestingly, an influence of the food matrix on the oral bioavailability of STX was noticed in mice, for which the NOAEL upon gavage appears to be about 2-fold lower compared to that obtained in mice fed with a mixture of cream cheese and toxin (Table S5), and it also took longer for the signs of intoxication to occur via feeding mixtures than via gavage.^49^ In a real-life scenario, acute STX poisoning is usually caused by consuming contaminated shellfish, and to what degree the presence of the food matrix will affect the bioavailability of STX in humans is still unknown. Fa and ka are influential model parameters (Figure S2). This indicates that factors such as administration patterns and fasting or feeding status of the animal upon dosing could affect STX absorption and model predictions and then have impacts on the further model-based QIVIVE and POD derivation. Our model predictions for rat urinary STX amounts and human blood STX concentrations match available data well (Figures 3 and 4), indicating that the current assumptions made for Fa and ka are adequate.

No substantial turnover of STX was observed in the rat hepatocyte assay (Figure S1), which is consistent with a finding where no detectable biotransformation was found when incubating GTX2/3 and C1/2 toxins (*N*-sulfo-carbamoyl analogues of STX) with liver enzyme preparations from rats and mice.^50^ Suspected metabolism of STX and its analogues in the human body was reported in several studies, based on the different toxin profiles observed between the causative foods and human biological specimens.^3^ Though first identified in 1957,^51^ the biotransformation of STX-group toxins is still unclear and might require further studies, although the results of the present study corroborate that STX metabolism may be limited and not a main contributor to its clearance from the systemic circulation.

Glomerular filtration was found to be a primary elimination way for STX in cats under normal cardiovascular conditions and diuresis,^10^ which is in line with the observation in the present study (Figure 3). At higher dose levels in cats, vascular hypotension induced by STX would reduce the glomerular filtration rate, decreasing its renal clearance.^10^ In human poisoning cases, both hypertension and hypotension caused by STX exposure were reported,^7^ and the changed blood pressure may affect the urinary excretion of STX, which might be an explanation for the discrepancy in the reported urinary STX concentrations between individuals at similar dose levels.^34^

The *in vivo* dose–response curves converted from available *in vitro* concentration–response datasets resulted in conservative POD predictions (Figure 6). A probable explanation is that the impacts of STX on the overall functioning of the neuronal network and the organism (i.e., lethality) might not be fully captured by assessing a single endpoint.^52^ Integration of PBK modeling-facilitated QIVIVE and the adverse outcome pathway (AOP) approach is a promising way to cover the toxicokinetic and toxicodynamic processes in the human body, as the AOP approach describes the entire toxicity pathway from the molecular initiating event (MIE) to the ultimate toxic effects (adverse outcome) at an organism level.^52,53^ STX prevents the conduction of cellular action potentials by blocking voltage-gated sodium channels.^3,13^ Data from *in vitro* STX toxicity assays (Tables S2–S4), especially the ones detecting electrophysiological signal inhibition on sodium ion channels, could be used as the MIE for the AOP. Then, the subsequent key events at organelle, cellular, tissue, organ, and organism levels could be linked, but further studies are still required to clarify the events and the relationships between them.

Humans are found to be more susceptible to the acute toxicity caused by STX exposure than rodents (Figure 6). The sodium ion channels are highly conserved in mammalian species,^54^ and STX shows roughly equipotency toward rat and human Nav1.4 expressed in stably transfected HEK-293 cells with the IC_50_ of 1.17 and 1.88 nM for rNav1.4 and hNav1.4, respectively.^2,37^ Given this information, the interspecies differences in STX toxicity between rodents and humans are assumed to be mainly due to differences in toxicokinetics. At the same dose levels, higher blood STX concentrations are predicted in humans than rodents (Figure S3), and one probable reason could be the higher glomerular filtration rates of mice and rats than humans, resulting in a faster renal clearance of STX in rodents.^33^ Using four sensitive sodium ion channel subtypes, the mean predicted human BMDL_10_ is within 2.8-fold lower than the reported human NOAELs.^3,13^ However, the impact of uncertainties from several sources on current risk assessment of the dietary exposure to STX-group toxins cannot be ignored, as the uncertainties in estimated exposure from the intoxication data, the usage of TEFs obtained in mice following intraperitoneal injection, and analytical methods used for sample analysis might have potentials to cause over- or underestimation of exposure and resulting risk.^3^

Although the currently developed PBK modeling-facilitated QIVIVE approach is promising to be used in future risk assessment, more information on toxicokinetics and toxicodynamics of STX is required for its further improvement and refinement. STX was found to be able to readily pass through the blood–brain barrier and was detected in brains of cats and rats after iv and intraperitoneal injections, respectively.^10,55^ In humans, STX was reported in the brain after post-mortem analysis of samples from two victims who were intoxicated by consumption of shellfish contaminated with STX-group toxins.^56^ However, detailed information about brain penetration of STX is not available, and the acute toxic effects following STX exposure are mostly peripheral, while its influence on the central nervous system has yet to be confirmed.^3,7^ Thus, the brain was not included as a separate compartment in the current PBK models but combined into the rapidly perfused tissues, and the maximum STX concentration in blood was used for the following QIVIVE. Since the predictions made especially for humans were in line with the actually reported PODs (Figure 6), it appears that this assumption provides an adequate first-tier approach. Studying STX transport in *in vitro* models for the blood–brain barrier may prove a way forward to define parameters for blood–brain transport that may be used to further refine the models.

Altogether, the results obtained provide a proof of principle for the use of the PBK modeling-facilitated QIVIVE approach, as an alternative to animal testing, for toxicity prediction of a phycotoxin. Exposure to phycotoxins might be a potential public health issue in the future due to the probable influence that the current climate trends might have on expanding geographic distribution of the toxigenic microalgae and the resulting phycotoxins. Thus, this study might shed light on the risk assessment work for other phycotoxins.

## Supporting Information Available

Materials and methods for the rat hepatocyte incubation assay; local sensitivity analysis for PBK model evaluation; summary of physiological and physicochemical parameters used for PBK models; summary of available *in vitro* toxicity studies of STX on rats, mice, and humans and corresponding predictions; summary of reported rodent and human POD values; PBK model codes for rats, mice, and humans (PDF)

## References

[ref1] World Health Organization Toxicity equivalency factors for marine biotoxins associated with bivalve molluscs; Technical paper; Joint Food and Agriculture Organization of the United Nations/World Health Organization: Rome, Italy, 2016; https://apps.who.int/iris/bitstream/handle/10665/250663/9789241511483-eng.pdf?sequence=1&isAllowed=y.

[ref2] Alonso E.; Alfonso A.; Vieytes M. R.; Botana L. M. Evaluation of toxicity equivalent factors of paralytic shellfish poisoning toxins in seven human sodium channels types by an automated high throughput electrophysiology system. Arch. Toxicol. 2016, 90, 479–488. 10.1007/s00204-014-1444-y. 25572188

[ref3] Scientific Opinion of the Panel on Contaminants in the Food Chain on a request from the European Commission on Marine Biotoxins in Shellfish – Saxitoxin Group. EFSA J. 2009, 1019, 1–76.

[ref4] Barrientos R. G.; Hernández-Mora G.; Alegre F.; Field T.; Flewelling L.; McGrath S.; Deeds J.; Chacón Y. S.; Rojas Arrieta K.; Vargas E. C.; Artavia K. B.; Stacy B. A. Saxitoxin poisoning in green turtles (*Chelonia mydas*) linked to scavenging on mass mortality of Caribbean sharpnose puffer fish (*Canthigaster rostrata*-Tetraodontidae). Front. Vet. Sci. 2019, 6, 466 10.3389/fvets.2019.00466. 31921922 PMC6928104

[ref5] McIntyre L.; Miller A.; Kosatsky T. Changing trends in paralytic shellfish poisonings reflect increasing sea surface temperatures and practices of indigenous and recreational harvesters in British Columbia, Canada. Mar. Drugs 2021, 19, 568 10.3390/md19100568. 34677468 PMC8538720

[ref6] Nicolas J.; Hoogenboom R. L. A. P.; Hendriksen P. J. M.; Bodero M.; Bovee T. F. H.; Rietjens I. M. C. M.; Gerssen A. Marine biotoxins and associated outbreaks following seafood consumption: Prevention and surveillance in the 21st century. Global Food Secur. 2017, 15, 11–21. 10.1016/j.gfs.2017.03.002.

[ref7] World Health Organization Cyanobacterial toxins: saxitoxins; Background document for development of WHO, guidelines for drinking-water quality and guidelines for safe recreational water environments; World Health Organization: Geneva, Switzerland, 2020; https://apps.who.int/iris/bitstream/handle/10665/338069/WHO-HEP-ECH-WSH-2020.8-eng.pdf?sequence=1&isAllowed=y.

[ref8] García C.; Barriga A.; Díaz J. C.; Lagos M.; Lagos N. Route of metabolization and detoxication of paralytic shellfish toxins in humans. Toxicon 2010, 55, 135–144. 10.1016/j.toxicon.2009.07.018. 19632259

[ref9] Stafford R. G.; Hines H. B. Urinary elimination of saxitoxin after intravenous injection. Toxicon 1995, 33, 1501–1510. 10.1016/0041-0101(95)00081-V. 8744989

[ref10] Andrinolo D.; Michea L. F.; Lagos N. Toxic effects, pharmacokinetics and clearance of saxitoxin, a component of paralytic shellfish poison (PSP), in cats. Toxicon 1999, 37, 447–464. 10.1016/S0041-0101(98)00173-1. 10080350

[ref11] Gessner B. D.; Bell P.; Doucette G. J.; Moczydlowski E.; Poli M. A.; Van Dolah F.; Hall S. Hypertension and identification of toxin in human urine and serum following a cluster of mussel-associated paralytic shellfish poisoning outbreaks. Toxicon 1997, 35, 711–722. 10.1016/S0041-0101(96)00154-7. 9203296

[ref12] Le Drean Y.; Bounouh A.; Douki T.; Dejardin O.; Demarquoy J.; Lacour B.; Thuroczy G.; Ducimetiere P.; Dore J.F.; Feltin N.; Gaffet E. Opinion of the French Agency for Food, Environmental and Occupational Health and Safety (ANSES) on the proposed acute oral TRV for saxitoxin; French Agency for Food, Environmental and Occupational Health and Safety: Maisons-Alfort, France, 2020; https://www.anses.fr/en/system/files/VSR2016SA0299EN.pdf.

[ref13] Toyofuku H. Report of the Joint FAO/IOC/WHO ad hoc Expert Consultation on biotoxins in bivalve molluscs; Program and meeting document; Joint Intergovernmental Oceanographic Commission/Food and Agriculture Organization of the United Nations/World Health Organization: Oslo, Norway, 2004; https://www.fao.org/3/au629e/au629e.pdf.

[ref14] Dent M. P.; Vaillancourt E.; Thomas R. S.; Carmichael P. L.; Ouedraogo G.; Kojima H.; Barroso J.; Ansell J.; Barton-Maclaren T. S.; Bennekou S. H.; Boekelheide K.; Ezendam J.; Field J.; Fitzpatrick S.; Hatao M.; Kreiling R.; Lorencini M.; Mahony C.; Montemayor B.; Mazaro-Costa R.; Oliveira J.; Rogiers V.; Smegal D.; Taalman R.; Tokura Y.; Verma R.; Willett C.; Yang C. Paving the way for application of next generation risk assessment to safety decision-making for cosmetic ingredients. Regul. Toxicol. Pharmacol. 2021, 125, 105026 10.1016/j.yrtph.2021.105026. 34389358 PMC8547713

[ref15] Louisse J.; Beekmann K.; Rietjens I. M. C. M. Use of physiologically based kinetic modeling-based reverse dosimetry to predict *in vivo* toxicity from *in vitro* data. Chem. Res. Toxicol. 2017, 30, 114–125. 10.1021/acs.chemrestox.6b00302. 27768849

[ref16] Rietjens I. M. C. M.; Louisse J.; Punt A. Tutorial on physiologically based kinetic modeling in molecular nutrition and food research. Mol. Nutr. Food Res. 2011, 55, 941–956. 10.1002/mnfr.201000655. 21520492

[ref17] Wang X.; Zhao X.; Shi D.; Dong Z.; Zhang X.; Liang W.; Liu L.; Wang X.; Wu F. Integrating physiologically based pharmacokinetic modeling-based forward dosimetry and *in vitro* bioassays to improve the risk assessment of organophosphate esters on human health. Environ. Sci. Technol. 2023, 57, 1764–1775. 10.1021/acs.est.2c04576. 36591971

[ref18] Chen Q.; Chou W. C.; Lin Z. Integration of toxicogenomics and physiologically based pharmacokinetic modeling in human health risk assessment of perfluorooctane sulfonate. Environ. Sci. Technol. 2022, 56, 3623–3633. 10.1021/acs.est.1c06479. 35194992

[ref19] Karrer C.; de Boer W.; Delmaar C.; Cai Y.; Crépet A.; Hungerbühler K.; von Goetz N. Linking probabilistic exposure and pharmacokinetic modeling to assess the cumulative risk from the bisphenols BPA, BPS, BPF, and BPAF for Europeans. Environ. Sci. Technol. 2019, 53, 9181–9191. 10.1021/acs.est.9b01749. 31294980

[ref20] Noorlander A.; Zhang M.; van Ravenzwaay B.; Rietjens I. M. C. M. Use of physiologically based kinetic modeling-facilitated reverse dosimetry to predict *in vivo* acute toxicity of tetrodotoxin in rodents. Toxicol. Sci. 2022, 187, 127–138. 10.1093/toxsci/kfac022. 35218365 PMC9041554

[ref21] Stafford R. G.; Hines H. B. Method for the identification of saxitoxin in rat urine. J. Chromatogr., B: Biomed. Sci. Appl. 1994, 657, 119–124. 10.1016/0378-4347(94)80077-4. 7952057

[ref22] Torres R.; Pizarro L.; Csendes A.; García C.; Lagos N. GTX 2/3 epimers permeate the intestine through a paracellular pathway. J. Toxicol. Sci. 2007, 32, 241–248. 10.2131/jts.32.241. 17785941

[ref23] Punt A.; Louisse J.; Pinckaers N.; Fabian E.; van Ravenzwaay B. Predictive performance of next generation physiologically based kinetic (PBK) model predictions in rats based on *in vitro* and *in silico* input data. Toxicol. Sci. 2022, 186, 18–28. 10.1093/toxsci/kfab150. 34927682 PMC8883350

[ref24] Punt A.; Louisse J.; Beekmann K.; Pinckaers N.; Fabian E.; van Ravenzwaay B.; Carmichael P. L.; Sorrell I.; Moxon T. E. Predictive performance of next generation human physiologically based kinetic (PBK) models based on *in vitro* and *in silico* input data. Altex 2022, 39, 221–234. 10.14573/altex.2108301. 35064272

[ref25] Pires D. E. V.; Blundell T. L.; Ascher D. B. pkCSM: Predicting small-molecule pharmacokinetic and toxicity properties using graph-based signatures. J. Med. Chem. 2015, 58, 4066–4072. 10.1021/acs.jmedchem.5b00104. 25860834 PMC4434528

[ref26] Sun D.; Lennernas H.; Welage L. S.; Barnett J. L.; Landowski C. P.; Foster D.; Fleisher D.; Lee K. D.; Amidon G. L. Comparison of human duodenum and Caco-2 gene expression profiles for 12,000 gene sequences tags and correlation with permeability of 26 drugs. Pharm. Res. 2002, 19, 1400–1416. 10.1023/A:1020483911355. 12425456

[ref27] Yu L. X.; Amidon G. L. A compartmental absorption and transit model for estimating oral drug absorption. Int. J. Pharm. 1999, 186, 119–125. 10.1016/S0378-5173(99)00147-7. 10486429

[ref28] Hong B.; Sun J.; Zheng H.; Le Q.; Wang C.; Bai K.; He J.; He H.; Dong Y. Effect of tetrodotoxin pellets in a rat model of postherpetic neuralgia. Mar. Drugs 2018, 16, 195 10.3390/md16060195. 29874779 PMC6025269

[ref29] Punt A.; Pinckaers N.; Peijnenburg A.; Louisse J. Development of a web-based toolbox to support quantitative *in-vitro*-to-*in-vivo* extrapolations (QIVIVE) within nonanimal testing strategies. Chem. Res. Toxicol. 2021, 34, 460–472. 10.1021/acs.chemrestox.0c00307. 33382582 PMC7887804

[ref30] Rogers R. S.; Rapoport H. The pKa’s of saxitoxin. J. Am. Chem. Soc. 1980, 102, 7335–7339. 10.1021/ja00544a030.

[ref31] Simcyp prediction tools – fu, https://members.simcyp.com/account/tools/fuc/.

[ref32] Cubitt H. E.; Houston J. B.; Galetin A. Relative importance of intestinal and hepatic glucuronidation—Impact on the prediction of drug clearance. Pharm. Res. 2009, 26, 1073–1083. 10.1007/s11095-008-9823-9. 19184618

[ref33] Walton K.; Dorne J. L. C. M.; Renwick A. G. Species-specific uncertainty factors for compounds eliminated principally by renal excretion in humans. Food Chem. Toxicol. 2004, 42, 261–274. 10.1016/j.fct.2003.09.001. 14667472

[ref34] Knaack J. S.; Porter K. A.; Jacob J. T.; Sullivan K.; Forester M.; Wang R. Y.; Trainer V. L.; Morton S.; Eckert G.; McGahee E.; Thomas J.; McLaughlin J.; Johnson R. C. Case diagnosis and characterization of suspected paralytic shellfish poisoning in Alaska. Harmful Algae 2016, 57, 45–50. 10.1016/j.hal.2016.03.006. 28918891

[ref35] McLaughlin J. B.; Fearey D. A.; Esposito T. A.; Porter K. A. Paralytic shellfish poisoning in Southeast Alaska, May–June 2011; Bulletin; State of Alaska Epidemiology: Anchorage, Alaska, 2011; http://www.epi.alaska.gov/bulletins/docs/b2011_17.pdf.

[ref36] Nicolas J.; Hendriksen P. J. M.; van Kleef R. G. D. M.; de Groot A.; Bovee T. F. H.; Rietjens I. M. C. M.; Westerink R. H. S. Detection of marine neurotoxins in food safety testing using a multielectrode array. Mol. Nutr. Food Res. 2014, 58, 2369–2378. 10.1002/mnfr.201400479. 25266399

[ref37] Vélez P.; Sierralta J.; Alcayaga C.; Fonseca M.; Loyola H.; Johns D. C.; Tomaselli G. F.; Marbán E.; Suárez-Isla B. A. A functional assay for paralytic shellfish toxins that uses recombinant sodium channels. Toxicon 2001, 39, 929–935. 10.1016/S0041-0101(00)00230-0. 11223080

[ref38] Kulagina N. V.; O’Shaughnessy T. J.; Ma W.; Ramsdell J. S.; Pancrazio J. J. Pharmacological effects of the marine toxins, brevetoxin and saxitoxin, on murine frontal cortex neuronal networks. Toxicon 2004, 44, 669–676. 10.1016/j.toxicon.2004.07.023. 15501293

[ref39] Perez S.; Vale C.; Botana A. M.; Alonso E.; Vieytes M. R.; Botana L. M. Determination of toxicity equivalent factors for paralytic shellfish toxins by electrophysiological measurements in cultured neurons. Chem. Res. Toxicol. 2011, 24, 1153–1157. 10.1021/tx200173d. 21619049

[ref40] Nicholson R. A.; Li G. H.; Buenaventura E.; Graham D. A rapid and sensitive assay for paralytic shellfish poison (PSP) toxins using mouse brain synaptoneurosomes. Toxicon 2002, 40, 831–838. 10.1016/S0041-0101(02)00083-1. 12175621

[ref41] Llewellyn L.; Negri A.; Quilliam M. High affinity for the rat brain sodium channel of newly discovered hydroxybenzoate saxitoxin analogues from the dinoflagellate *Gymnodinium catenatum*. Toxicon 2004, 43, 101–104. 10.1016/j.toxicon.2003.10.016. 15037035

[ref42] Melegari S. P.; de Carvalho Pinto C. R. S.; Moukha S.; Creppy E. E.; Matias W. G. Evaluation of cytotoxicity and cell death induced *in vitro* by saxitoxin in mammalian cells. J. Toxicol. Environ. Health, Part A 2015, 78, 1189–1200. 10.1080/15287394.2015.1072069. 26436995

[ref43] Arnich N.; Thébault A. Dose-response modelling of paralytic shellfish poisoning (PSP) in humans. Toxins 2018, 10, 141 10.3390/toxins10040141. 29597338 PMC5923307

[ref44] Huang Foen Chung J. W. N. C.; van Mastrigt R. Age and volume dependent normal frequency volume charts for healthy males. J. Urol. 2009, 182, 210–214. 10.1016/j.juro.2009.02.113. 19447412

[ref45] Munday R.; Thomas K.; Gibbs R.; Murphy C.; Quilliam M. A. Acute toxicities of saxitoxin, neosaxitoxin, decarbamoyl saxitoxin and gonyautoxins 1&4 and 2&3 to mice by various routes of administration. Toxicon 2013, 76, 77–83. 10.1016/j.toxicon.2013.09.013. 24060374

[ref46] Finch S. C.; Boundy M. J.; Harwood D. T. The acute toxicity of tetrodotoxin and tetrodotoxin–saxitoxin mixtures to mice by various routes of administration. Toxins 2018, 10, 423 10.3390/toxins10110423. 30360529 PMC6266834

[ref47] Nguyen H. V. N.; Smith M. E.; Swoboda H. D. Shellfish toxicity (Last update: July 18, 2022); https://www.ncbi.nlm.nih.gov/books/NBK470225/.

[ref48] Suarez-Isla B. A. Saxitoxin and other paralytic toxins: toxicological profile. In Marine and Freshwater Toxins; Gopalakrishnakone P.; Haddad V. Jr.; Tubaro A.; Kim E.; Kem W. Eds.; Springer: Dordrecht, the Netherlands, 2016; pp. 23.

[ref49] Selwood A. I.; Waugh C.; Harwood D. T.; Rhodes L. L.; Reeve J.; Sim J.; Munday R. Acute toxicities of the saxitoxin congeners gonyautoxin 5, gonyautoxin 6, decarbamoyl gonyautoxin 2&3, decarbamoyl neosaxitoxin, C-1&2 and C-3&4 to mice by various routes of administration. Toxins 2017, 9, 73 10.3390/toxins9020073. 28230783 PMC5331452

[ref50] Hong H.; Lam P. K. S.; Hsieh D. P. H. Interactions of paralytic shellfish toxins with xenobiotic-metabolizing and antioxidant enzymes in rodents. Toxicon 2003, 42, 425–431. 10.1016/S0041-0101(03)00175-2. 14505944

[ref51] Wiese M.; D’Agostino P. M.; Mihali T. K.; Moffitt M. C.; Neilan B. A. Neurotoxic alkaloids: Saxitoxin and its analogs. Mar. Drugs 2010, 8, 2185–2211. 10.3390/md8072185. 20714432 PMC2920551

[ref52] Kasteel E. E. J.; Westerink R. H. S. Refining *in vitro* and *in silico* neurotoxicity approaches by accounting for interspecies and interindividual differences in toxicodynamics. Expert Opin. Drug Metab. Toxicol. 2021, 17, 1007–1017. 10.1080/17425255.2021.1885647. 33586568

[ref53] Villeneuve D. L.; Crump D.; Garcia-Reyero N.; Hecker M.; Hutchinson T. H.; LaLone C. A.; Landesmann B.; Lettieri T.; Munn S.; Nepelska M.; Ottinger M. A.; Vergauwen L.; Whelan M. Adverse outcome pathway (AOP) development I: Strategies and principles. Toxicol. Sci. 2014, 142, 312–320. 10.1093/toxsci/kfu199. 25466378 PMC4318923

[ref54] Kasteel E. E. J.; Westerink R. H. S. Comparison of the acute inhibitory effects of tetrodotoxin (TTX) in rat and human neuronal networks for risk assessment purposes. Toxicol. Lett. 2017, 270, 12–16. 10.1016/j.toxlet.2017.02.014. 28192153

[ref55] Cianca R. C. C.; Pallares M. A.; Barbosa R. D.; Adan L. V.; Martins J. M. L.; Gago-Martínez A. Application of precolumn oxidation HPLC method with fluorescence detection to evaluate saxitoxin levels in discrete brain regions of rats. Toxicon 2007, 49, 89–99. 10.1016/j.toxicon.2006.09.021. 17097704

[ref56] García C.; del Carmen Bravo M.; Lagos M.; Lagos N. Paralytic shellfish poisoning: post-mortem analysis of tissue and body fluid samples from human victims in the Patagonia fjords. Toxicon 2004, 43, 149–158. 10.1016/j.toxicon.2003.11.018. 15019474

