# Supplementary material for: Integrating In Vitro Data and Physiologically Based Kinetic Modeling to Predict and Compare Acute Neurotoxic Doses of Saxitoxin in Rats, Mice, and Humans
Source: Environ Sci Technol. 2023 Jul 21;57(30):10974–84. doi: 10.1021/acs.est.3c01987 (PMC10399293; doi:10.1021/acs.est.3c01987)
Supplement: Supplementary file 1 — es3c01987_si_001.pdf [file es3c01987_si_001.pdf]

SUPPORTING INFORMATION

Integrating *In Vitro* Data and Physiologically Based Kinetic Modeling to Predict and Compare Acute Neurotoxic Doses of Saxitoxin in Rats, Mice, and Humans

Jiaqi Chen[a], Annelies Noorlander[a], Sebastiaan Wesseling[a], Hans Bouwmeester[a], Nynke I. Kramer[a], Ivonne M.C.M. Rietjens[a]*

[a] Division of Toxicology, Wageningen University and Research, Stippeneng 4, 6708 WE, Wageningen, The Netherlands

[*] Corresponding author: Jiaqi Chen (Email: jiaqi.chen@wur.nl)

22 Pages, 3 Figures, 5 Tables

Supplementary Materials and Method

Chemicals and biological materials

A certified calibration solution for saxitoxin dihydrochloride (CRM-STX-g, 61.4 ± 2.4 µM in aqueous 0.5 mM hydrochloric acid) was purchased from the National Research Council, Institute for Marine Biosciences (Halifax, Canada). Primary pooled rat cryopreserved hepatocytes (male Sprague-Dawley rats), hepatocyte thawing/plating supplement pack (CM3000), cell maintenance supplement pack (CM4000), and Williams' Medium E (1×, no phenol red) were obtained from Thermo Fisher (Landsmeer, the Netherlands). Formic acid, testosterone, and cell proliferation reagent WST-1 were purchased from Sigma-Aldrich (St. Louis, MO, USA). Acetonitrile (ACN, UPLC/MS grade) and methanol (UPLC/MS grade) were bought from Biosolve (Valkenswaard, the Netherlands). Thawing medium and incubation medium were prepared with CM3000, CM4000 and Williams' Medium E as recommended by the guidelines of the supplier (Thermo Fisher 2011). Briefly, 25 mL fetal bovine serum, 50 µL dexamethasone in DMSO and 18 mL Cocktail-A in CM3000 pack were added to 500 mL Williams' Medium E as the thawing medium; and 5 µL dexamethasone in DMSO and 20 mL Cocktail-B in CM4000 pack were added to 500 mL Williams' Medium E as the incubation medium. The media were stored at 4℃ and pre-warmed to 37℃ before using. Ultrapure water passed through a Sartorius Arium® Pro ultrapure water system (Göttingen, Germany) was used during experiments.

Rat hepatocyte incubation assay

Cryopreserved hepatocytes were shortly thawed in a 37℃ water bath, then immediately transferred to 50 mL thawing medium and centrifuged at 60×g for 5 min at room temperature. After removing the supernatant, the hepatocytes were re-suspended in 1 mL pre-warmed incubation medium. Hepatocyte density and viability were measured using Cellometer (Auto T4, Nexcelom Bioscience, Lawrence, MA, USA), after which the cells (viability >90%) were further diluted to 1×10^6 viable cells/mL with incubation medium. The commercially available STX solution was diluted with incubation medium to 2 µM as the working solution, and then 100 µL of this solution was added to each well of 96-well flat-bottom transparent plates (Greiner Bio-One, the Netherlands). After 5-min pre-incubation, the incubation was started by adding 100 µL hepatocyte suspension to each well, giving a final concentration of 0.5×10^6 cells/mL and 1 µM STX. Plates were put on a shaker (Polymax 1040, Heidolph, Germany) at 50 rpm in a 5% CO_2, 95% air-humidified incubator (37℃). After incubating for 0, 5, 15, 30, 45, 60, 90 and 120 min, the reaction was terminated by transferring 100 µL well-mixed liquid from each well to a tube containing 50 µL ice-cold ACN, and the resulting mixture was vortexed and then kept on ice for 15 min. Controls of each timepoint were carried out by replacing hepatocyte suspension with incubation medium only. After centrifuging at 15000×g for 5 min at 4℃, the remaining STX in the supernatants was quantified by LC-MS/MS. Hepatocyte incubations with testosterone (final

concentration of 1 μM in incubation medium containing 0.2% methanol) were used as positive control. Two independent experiments were preformed and each included three replicates.

Cytotoxicity of 1 μM STX to rat hepatocytes was evaluated by the WST-1 assay. Briefly, 5 μL WST-1 regent was added to the remaining 100 μL liquid in each well of the 96-well plates after STX incubation, and incubated for 2 hours in a 5% CO_2, 95% air-humidified incubator (37°C). The absorbance was measured at 440 nm and 620 nm (subtracted as background absorbance) using SpectraMax® iD3 (Molecular Devices, San Jose, CA, USA).

LC-MS/MS analysis

Quantification of STX was performed on a Shimadzu Nexera XR LC-20AD SR UPLC system connected to a Shimadzu LCMS-8045 triple quadrupole mass spectrometer (Kyoto, Japan). Chromatographic separation was conducted on a Waters Acquity UPLC BEH Amide analytical column (2.1 mm × 100 mm, 1.7 μm) coupled with a BEH Amide pre-column (2.1 mm × 5 mm, 1.7 μm). Column temperature was maintained at 40°C and the auto-sampler temperature at 10°C during analysis. Ultrapure water and ACN were used as mobile phases, both containing 0.1% (*v/v*) formic acid. Using a 1 μL injection volume, a 12-min linear gradient with a flow rate of 0.3 mL/min first ran from 5% to 50% water over 2 min, then returned to 5% water over 5 min and was held at this ratio for 5 min. The UPLC system was coupled with an electrospray ionization (ESI) interface to the mass spectrometer. The acquisition was performed in positive multiple reaction monitoring (MRM) mode, and precursor ($[M+H]^+$)/product ions monitored for STX were 300>282 (CE 19 eV), 300>204 (CE 25 eV) and 300>138 (CE 31 eV). The detection limit of STX was 0.5 nM. Quantification was based on a linear calibration curve ($r^2 > 0.99$) obtained from the peak area of the ion chromatogram of each standard solution prepared by diluting commercially available STX with a 2:1 (*v/v*) mixture of incubation medium and ACN. LabSolutions software (version 5.98, Kyoto, Japan) was used for instrument control, data acquisition and data processing.

Calculation of *in vitro* intrinsic clearance of STX by rat hepatocytes

The remaining concentration of STX in the samples was compared with STX in the sample at 0 min, and the natural logarithm of the percentage of the remaining substrate (Ln (Remaining % STX)) was plotted against time. The slope of the linear part of this depletion curve represents the elimination rate constant (k, in min^{-1}) (Yamagata et al. 2017). The *in vitro* intrinsic clearance ($CL_{int,\ in\ vitro}$, in μL/min/10^6 cells) of the substrate was subsequently calculated using the following equation (Eq S1):

$$CL_{int,in\ vitro} = \frac{k \times V}{n} \quad \text{(Eq S1)}$$

where V is the volume of the incubation mixture (200 μL), and n represents the number of hepatocytes in the incubation (0.1×10^6 viable cells). Data were collected from two independent experiments and each data point was presented as the mean value ± SEM using GraphPad Prism (version 5.04, San Diego, CA, USA).

Sensitivity analysis

A local sensitivity analysis was performed to identify the influential parameters on the predicted maximum blood STX concentration as a model output. The normalized sensitivity coefficients (SCs) were calculated with the following Equation S2 (Eq S2):

$$SC = \frac{(C' - C)}{(P' - P)} \times \frac{P}{C} \quad \text{(Eq S2)}$$

where *P* represents the original parameter value in the PBK model and *P'* is the parameter value with a 5% increase, *C* is the model output with the initial parameter values and *C'* is the parameter value after a 5% increase. Only the parameters with an absolute SC > 0.1 were considered to be influential on the model output (WHO 2010). The sensitivity analysis was carried out using an oral dose level of 0.163 mg/kg BW for the rat and mouse models, representing the NOAEL obtained in mice upon oral exposure (gavage) (Munday et al. 2013). For the human model, an oral dose level of 0.0005 mg/kg BW was used for the sensitivity analysis, representing the NOAEL derived by EFSA (EFSA 2009).

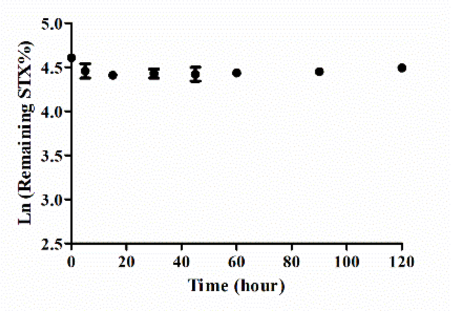

Figure S1 Time-dependent substrate depletion of STX in incubations with rat hepatocytes in suspension. The dots represent the natural logarithm of the percentage of the remaining STX (Ln(Remaining STX%)) at different incubation timepoints (mean ± SEM).

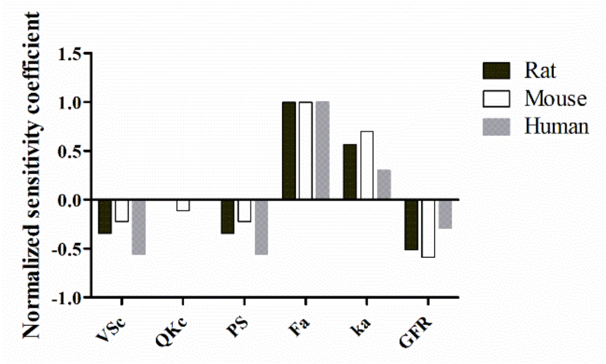

Figure S2 Sensitivity analysis for the predicted maximum blood STX concentration at an oral dose level of 0.163 mg/kg BW (rat and mouse), and an oral dose level of 0.0005 mg/kg BW (human). Model parameters with a normalized SC higher than 0.1 (absolute value) are shown, representing an influential value on the model output. VSc, fraction of slowly perfused tissue; QKc, fraction of blood flow to kidney; PS, slowly perfused tissue:blood partition coefficient of STX; Fa, fraction of dose absorbed; ka, absorption rate constant; GFR, glomerular filtration rate.

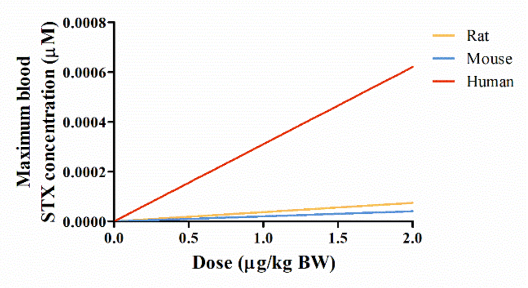

Figure S3 PBK modeling-based predictions of dose-dependent maximum blood STX concentration in rats, mice and humans.

Table S1 Summary of physiological and physicochemical parameters used for the PBK models as obtained from literature (Brown et al. 1997; Hall et al. 2012)

Model parameters	Symbols in model code	Rat	Mouse	Human
Physiological parameters				
Body weight (BW, kg)	BW	0.215	0.03	60
Percentage of body weight				
Liver	VLc	3.4	5.5	2.6
Fat	VFc	7.0	7.0	21.4
Kidney	VKc	0.7	1.7	0.4
Rapidly perfused tissue	VRc	9.1	13.8	6.4
Slowly perfused tissue	VSc	72.4	65.4	61.3
Blood	VBc	7.4	6.6	7.9
Cardiac output	QC	15.0[a]	16.5[b]	15.0[a]
Percentage of cardiac output				
Liver	QLc	17.4	15.8	22.7
Fat	QFc	7.0	7.0	5.2
Kidney	QKc	14.1	11.4	17.5
Rapidly perfused tissue	QRc	9.3	51.6	25.5
Slowly perfused tissue	QSc	52.2	14.2	29.1
Tissue: blood partition coefficients				
Liver	PL	2.88	2.88	2.88
Fat	PF	0.17	0.17	0.17
Kidney	PK	2.51	2.51	2.51
Rapidly perfused tissue	PR	2.71	2.80	2.80
Slowly perfused tissue	PS	2.91	2.87	3.04

a: L/hr/kg $BW^{0.74}$;
b: L/hr/kg $BW^{0.75}$.

Table S2 Summary of *in vitro* toxicity data of STX in rats and corresponding predicted $BMDL_{10}$ and BMD_{50} values using PBK modeling-facilitated reverse dosimetry

Endpoint	*In vitro* model	Detection method	IC_{50} (EC_{50}) (nM)	Reference	Predicted $BMDL_{10}$ (μg/kg BW)	Predicted BMD_{50} (μg/kg BW)
Electrophysiological signal inhibition	Rat neonatal cortical culture	Microelectrode array (MEA)	0.5 (Mean spike rate)	Nicolas et al. 2014	0.33	1.17
	HEK-293 cells transfected with rNav1.4	Patch-clamp current recordings	1.17	Vélez et al. 2001	1.09	3.22
	CHO cells transfected with rNav 1.4	Patch-clamp current recordings	1.8	Moran et al. 2003	2.21	11.38
	NGF-differentiated PC12 cells (rNav 1.2 and rNav 1.7)	Patch-clamp current recordings	2.1	Ondrus et al. 2012	3.98	17.12
	CHO cells transfected with rNav 1.4	Patch-clamp current recordings	2.8	Walker et al. 2012	1.34	7.88
	CHO cells transfected with rNav 1.4	Patch-clamp current recordings	2.9	Andresen and Bois. 2009	1.55	4.92
Receptor affinity	Rat brain synaptosomes	Radioactivity quantification	3.66	Doucette et al. 1997	8.69	31.95
	Rat brain synaptosomes	Radioactivity quantification	4.3	Llewellyn et al. 2004	6.34	21.31
	Receptors prepared from rat cerebral cortex	Radioactivity quantification	4.38	Usup et al. 2004	6.04	22.09

Table S3 Summary of *in vitro* toxicity data of STX in mice and corresponding predicted $BMDL_{10}$ and BMD_{50} values using PBK modeling-facilitated reverse dosimetry

**Endpoint**	***In vitro* model**	**Detection method**	**IC_{50} (EC_{50}) (nM)**	**Reference**	**Predicted $BMDL_{10}$ (μg/kg BW)**	**Predicted BMD_{50} (μg/kg BW)**
Electro-physiological signal inhibition	Cultured frontal cortex neuronal networks	Microelectrode array (MEA)	Number of spikes per burst: 1.2<br>Burst duration: 1.7<br>Mean spike rate: 1.8<br>Burst rate: 2.2	Kulagina et al. 2004	Number of spikes per burst: 2.84<br>Burst duration: 6.01<br>Mean spike rate: 29.22<br>Burst rate: 37.51	Number of spikes per burst: 8.13<br>Burst duration: 23.32<br>Mean spike rate: 43.79<br>Burst rate: 63.58
	Primary cultures of mouse cerebellar neurons (cerebellar granule cells)	Electro-physiological recordings in voltage-clamp mode	3.59	Perez et al. 2011	24.83	70.99
	Mouse brain synaptoneurosomes	Rhodamine 6G fluorescence quantification	4	Nicholson et al. 2002	23.61	46.29
	Primary cultures of mouse cerebellar neurons (cerebellar granule cells)	Fluorescence quantification	5.9	Vale et al. 2008	13.58	45.45
Cytotoxicity	Neuro-2a cell (w/o ouabain/veratridine)	MTT assay	1.01	Melegari et al. 2015	12.33	30.89
	Neuro-2a cell (w/o ouabain/veratridine)	MTT assay	1.5	Perreault et al. 2011	15.80	35.14
	Neuro-2a cell (with ouabain/veratridine)	MTT assay	8.0	Viallon et al. 2020	44.69	124.37
	Neuro-2a cell (with ouabain/veratridine)	MTT assay	8.5	Aballay-González et al. 2020	71.39	141.47
	Neuro-2a cell (with ouabain/veratridine)	MTT assay	8.6	Cañete and Dioène. 2008	23.38	72.83

Table S4 Summary of *in vitro* toxicity data of STX in humans and corresponding predicted $BMDL_{10}$ and BMD_{50} values using PBK modeling-facilitated reverse dosimetry

Endpoint	*In vitro* model	Detection method	IC_{50} (EC_{50}) (nM)	Reference	Predicted $BMDL_{10}$ (μg/kg BW)	Predicted BMD_{50} (μg/kg BW)
Electrophysiological signal inhibition	HEK-293 cell line transfected with hNav 1.1, 1.2, 1.3, 1.4, 1.5, 1.6, and 1.7	Automated Patch clamp electro-physiological recordings	hNav 1.2: 1<br>hNav 1.6: 1.09<br>hNav 1.4: 1.88<br>hNav 1.1: 2.3<br>hNav 1.3: 13.4<br>hNav 1.5: 212.6<br>hNav 1.7: 408	Alonso et al. 2016	hNav 1.2: 0.09<br>hNav 1.6: 0.30<br>hNav 1.4: 0.31<br>hNav 1.1: 0.30<br>hNav 1.3: 1.91<br>hNav 1.5: 157.1<br>hNav 1.7: 188.5	hNav 1.2: 2.03<br>hNav 1.6: 1.41<br>hNav 1.4: 1.22<br>hNav 1.1: 0.94<br>hNav 1.3: 8.35<br>hNav 1.5: 288.9<br>hNav 1.7: 727.1
	CHO cells transfected with hNav 1.5	Patch-clamp current recordings	256	Hoehne et al. 2013	26.20	81.16
	CHO cells transfected with hNav 1.7	Patch-clamp current recordings	702	Walker et al. 2012	49.29	152.4

Table S5 Reported rodent and human NOAEL and LD_{50} values upon acute oral exposure to STX

POD (μg/kg BW)	Mouse	Rat	Human
**Reported NOAEL**	163 (gavage)[a]	-[c]	0.5[d]
	341 (feeding)[b]		0.7[e]
**Reported LD_{50}**	356, 370 (gavage)[a,b]	192-212[d]	5.7[f]
	853, 958 (feeding)[a,b]		16.7-66.7[g]

a: Munday et al. (2013);
b: Finch et al. (2018);
c: no data available;
d: EFSA (2009);
e: FAO/IOC/WHO (2004);
f: Nguyen et al. (2021);
g: Suarez-Isla. (2016).

PBK model code

Rat model

```
;Date: Dec-9-2022
;Purpose: PBK model Saxitoxin (STX), built with in vitro and in silico derived parameters
;Species: Rat
;Compiled by: Jiaqi Chen and Annelies Noorlander
;Organization: Wageningen University

;=====================================================================
;Physiological parameters
;=====================================================================
;tissue volumes (Brown et al. 1997)
BW = 0.215                  ; (kg), average rat body weight from in vivo kinetic study (Stafford and Hines, 1995)
VFc = 0.070                 ; fraction of fat tissue
VLc = 0.034                 ; fraction of liver tissue
VKc = 0.007                 ; fraction of kidney tissue
VBc = 0.074                 ; fraction of blood
VRc = 0.091                 ; fraction of rapidly perfused tissues
VSc = 0.724                 ; fraction of slowly perfused tissues
                            ; total fraction is 1
VF = VFc*BW                 ; (L or kg), volume of fat tissue (calculated)
VL = VLc*BW                 ; (L or kg), volume of liver tissue (calculated)
VK = VKc*BW                 ; (L or kg), volume of kidney tissue (calculated)
VB = VBc*BW                 ; (L or kg), volume of blood (calculated)
VR = VRc*BW                 ; (L or kg), volume of rapidly perfused tissues (calculated)
VS = VSc*BW                 ; (L or kg), volume of slowly perfused tissues (calculated)
;---------------------------------------------------------------------------------------------------------------------
;blood flow rates (Brown et al. 1997)
QC = 15*BW^0.74             ; (L/hr), cardiac output
QFc = 0.070                 ; fraction of blood flow to fat
QLc = 0.174                 ; fraction of blood flow to liver
QKc = 0.141                 ; fraction of blood flow to kidney
QRc = 0.093                 ; fraction of blood flow to rapidly perfused tissues
QSc = 0.522                 ; fraction of blood flow to slowly perfused tissues
                            ; total fraction is 1
QF = QFc*QC                 ; (L/hr), blood flow to fat tissue (calculated)
QL = QLc*QC                 ; (L/hr), blood flow to liver tissue (calculated)
QK = QKc*QC                 ; (L/hr), blood flow to kidney tissue (calculated)
QR = QRc*QC                 ; (L/hr), blood flow to rapidly perfused tissues (calculated)
QS = QSc*QC                 ; (L/hr), blood flow to slowly perfused tissues (calculated)

;=====================================================================
;Partition Coefficients
;=====================================================================
;tissue/blood partition coefficients were obtained by dividing tissue/plasma partition coefficients (Rodgers and Rowland method, QIVIVE tool,
version 2.0, Punt et al. 2021) by corresponding blood/plasma ratio (BPr), which is assumed to be 1 for basic compounds (Cubitt et al. 2009)
;LogP  = -4.6               ; WHO (2020)
;pKa: 8.22, 11.28           ; Rogers and Rapoport. 1980
PF = 0.17                   ; fat/blood partition coefficient
PL = 2.88                   ; liver/blood partition coefficient
PK = 2.51                   ; kidney/blood partition coefficient
PR = 2.71                   ; rapidly perfused tissues/blood partition coefficient
PS = 2.91                   ; slowly perfused tissues/blood partition coefficient

;=====================================================================
;Kinetic parameters
;=====================================================================
;Transport from needle to blood
kn  =  1000000              ; (/hr), rate from needle to blood
;---------------------------------------------------------------------------------------------------------------------
;Fa=Fraction absorbed
Fa = 0.1                    ; calculated with QSAR-based equations
;Absorption constant
ka  = 0.07                  ; (/hr), calculated with QSAR-based equations
;---------------------------------------------------------------------------------------------------------------------
;Excretion from kidney via glomerular filtration
;Glomerular filtration
GFR = 5.2                                       ; (mL/min/kg bw), Walton et al., 2004
GF = GFR/1000*BW*60                             ; (L/hr), rat glomerular filtration rate

;=====================================================================
;Run settings
;=====================================================================
```

```
;Molecular weight
MW = 299.29                                     ; (g/mol), molecular weight STX (PubChem)

;Oral dose
ODOSEmg = 0                                     ; (mg/kg bw), given oral dose in mg/kg bw
ODOSEumol2 = ODOSEmg*1E-3/MW*1E6                ; (umol/kg bw), given oral dose recalculated to umol/kg bw
ODOSEumol=ODOSEumol2*BW                         ; (umol), given oral dose in umol
;--------------------------------------------------------------------------------------------------------------------
;Intravenous dose is 2 ug/kg bw = 0.002 mg/kg bw (Stafford and Hines, 1995)
IVDOSEmg = 0.002                                ; (mg/kg bw), given IV dose in mg/kg bw
IVDOSEumol2 = IVDOSEmg*1E-3/MW*1E6              ; (umol/kg bw), given IV dose recalculated to umol/kg bw
IVDOSEumol = IVDOSEumol2*BW                     ; (umol), given IV dose in umol

;time
Starttime = 0          ; in hr
Stoptime = 24          ; in hr

;======================================================================
;Model calculations
;======================================================================
;STX model
;--------------------------------------------------------------------------------------------------------------------
;needle compartment
;ANe = amount in needle, umol
ANe' = -kn*ANe
Init ANe = IVDOSEumol
;--------------------------------------------------------------------------------------------------------------------
;GI-tract compartment
;ASt  = amount of STX in GI-tract, umol
Ast'  = -ka*Ast
Init ASt =ODOSEumol*Fa
;--------------------------------------------------------------------------------------------------------------------
;liver compartment
;AL = amount of STX in liver tissue, umol
AL' = ka*ASt + QL*(CB - CVL)
Init AL = 0
CL = AL/VL
CVL = CL/PL
;--------------------------------------------------------------------------------------------------------------------
;kidney compartment
;AK =  amount of STX in kidney tissue, umol
AK' = QK*(CB-CVK) -AGF'
Init AK = 0
CK = AK/VK
CVK = CK/PK

;AGF = amount of STX cleared in urine via glomerular filtration, umol
AGF' = CVK*GF
Init AGF = 0
;--------------------------------------------------------------------------------------------------------------------
;fat compartment
;AF = amount of STX in fat tissue, umol
AF' = QF*(CB-CVF)
Init AF = 0
CF = AF/VF
CVF = CF/PF
;--------------------------------------------------------------------------------------------------------------------
;tissue compartment (rapidly perfused tissues)
;AR = amount of STX in rapidly perfused tissues, umol
AR' = QR*(CB-CVR)
Init AR = 0
CR = AR/VR
CVR = CR/PR
;--------------------------------------------------------------------------------------------------------------------
;tissue compartment (slowly perfused tissues)
;AS = amount of STX in slowly perfused tissues, umol
AS' = QS*(CB-CVS)
Init AS = 0
CS = AS/VS
CVS = CS/PS
;--------------------------------------------------------------------------------------------------------------------
;blood compartment
;AB = amount of STX in blood, umol
AB' = QF*CVF +  QL*CVL  + QK*CVK + QS*CVS + QR*CVR + kn*ANe - (QF+QL+QK+QS+QR)*CB
Init AB = 0
CB = AB/VB                               ; (umol/L), blood STX concentration
```

```
;====================================================================
;Mass balance calculations
;====================================================================
Total = IVDOSEumol + ODOSEumol * Fa
Calculated =  ANe + ASt + AL + AF + AK + AS + AR  + AB  + AGF

ERROR=((Total-Calculated)/Total+1E-30)*100
MASSBBAL=Total-Calculated + 1
```

Mouse model

```
;Date: Dec-9-2022
;Purpose: PBK model Saxitoxin (STX), built with in vitro and in silico derived parameters
;Species: Mouse
;Compiled by: Jiaqi Chen and Annelies Noorlander
;Organization: Wageningen University

;=====================================================================
;Physiological parameters
;=====================================================================
;tissue volumes (Hall et al. 2012)
BW = 0.03                     ; (kg), mouse body weight
VFc = 0.070                   ; fraction of fat tissue
VLc = 0.055                   ; fraction of liver tissue
VKc = 0.017                   ; fraction of kidney tissue
VBc = 0.066                   ; fraction of blood
VRc = 0.138                   ; fraction of rapidly perfused tissues
VSc = 0.654                   ; fraction of slowly perfused tissues
                              ; total fraction is 1
VF = VFc*BW                   ; (L or kg), volume of fat tissue (calculated)
VL = VLc*BW                   ; (L or kg), volume of liver tissue (calculated)
VK = VKc*BW                   ; (L or kg), volume of kidney tissue (calculated)
VB = VBc*BW                   ; (L or kg), volume of blood (calculated)
VR = VRc*BW                   ; (L or kg), volume of rapidly perfused tissues (calculated)
VS = VSc*BW                   ; (L or kg), volume of slowly perfused tissues (calculated)
;---------------------------------------------------------------------------------------------------------------
;blood flow rates (Hall et al. 2012)
QC = 16.5*BW^0.75             ; (L/hr), cardiac output (Brown et al. 1997)
QFc = 0.070                   ; fraction of blood flow to fat
QLc = 0.158                   ; fraction of blood flow to liver
QKc = 0.114                   ; fraction of blood flow to kidney
QRc = 0.516                   ; fraction of blood flow to rapidly perfused tissues
QSc = 0.142                   ; fraction of blood flow to slowly perfused tissues
                              ; total fraction is 1
QF = QFc*QC                   ; (L/hr), blood flow to fat tissue (calculated)
QL = QLc*QC                   ; (L/hr), blood flow to liver tissue (calculated)
QK = QKc*QC                   ; (L/hr), blood flow to kidney tissue (calculated)
QR = QRc*QC                   ; (L/hr), blood flow to rapidly perfused tissues (calculated)
QS = QSc*QC                   ; (L/hr), blood flow to slowly perfused tissues (calculated)

;=====================================================================
;Partition Coefficients
;=====================================================================
;tissue/blood partition coefficients were obtained by dividing tissue/plasma partition coefficients (Rodgers and Rowland method, QIVIVE tool, version 2.0, Punt et al. 2021) by corresponding blood/plasma ratio (BPr), which is assumed to be 1 for basic compounds (Cubitt et al. 2009)
;LogP  = -4.6                 ; WHO (2020)
;pKa: 8.22, 11.28             ; Rogers and Rapoport. 1980
PF = 0.17                     ; fat/blood partition coefficient
PL = 2.88                     ; liver/blood partition coefficient
PK = 2.51                     ; kidney/blood partition coefficient
PR = 2.80                     ; rapidly perfused tissues/blood partition coefficient
PS = 2.87                     ; slowly perfused tissues/blood partition coefficient

;=====================================================================
;Kinetic parameters
;=====================================================================
;Transport from needle to blood
kn  =  1000000                ; (/hr), rate from needle to blood
;---------------------------------------------------------------------------------------------------------------
;Fa=Fraction absorbed
Fa = 0.1                      ; calculated with QSAR-based equations
;Absorption constant
ka  = 0.07                    ; (/hr), calculated with QSAR-based equations
;---------------------------------------------------------------------------------------------------------------
;Excretion from kidney via glomerular filtration
;Glomerular filtration
GFR = 14                      ; (mL/min/kg bw), Walton et al., 2004
GF = GFR/1000*BW*60           ; (L/hr), mouse glomerular filtration rate

;=====================================================================
;Run settings
;=====================================================================
;Molecular weight
MW = 299.29                                            ; (g/mol), molecular weight STX (PubChem)
```

```
;Oral dose
ODOSEmg = 0                                            ; (mg/kg bw), given oral dose in mg/kg bw
ODOSEumol2 = ODOSEmg*1E-3/MW*1E6                       ; (umol/kg bw), given oral dose recalculated to umol/kg bw
ODOSEumol=ODOSEumol2*BW                                ; (umol), given oral dose in umol
;-------------------------------------------------------------------------------------------------------------------
;Intravenous dose is 2 ug/kg bw = 0.002 mg/kg bw (Stafford and Hines, 1995)
IVDOSEmg = 0.002                                       ; (mg/kg bw), given IV dose in mg/kg bw
IVDOSEumol2 = IVDOSEmg*1E-3/MW*1E6                     ; (umol/kg bw), given IV dose recalculated to umol/kg bw
IVDOSEumol = IVDOSEumol2*BW                            ; (umol), given IV dose in umol

;time
Starttime = 0          ; in hr
Stoptime = 24          ; in hr

;=====================================================================
;Model calculations
;=====================================================================
;STX model
;-------------------------------------------------------------------------------------------------------------------
;needle compartment
;ANe = amount in needle, umol
ANe' = -kn*ANe
Init ANe = IVDOSEumol
;-------------------------------------------------------------------------------------------------------------------
;GI-tract compartment
;ASt  = amount of STX in GI-tract, umol
Ast'  = -ka*Ast
Init ASt =ODOSEumol*Fa
;-------------------------------------------------------------------------------------------------------------------
;liver compartment
;AL = amount of STX in liver tissue, umol
AL' = ka*ASt + QL*(CB - CVL)
Init AL = 0
CL = AL/VL
CVL = CL/PL
;-------------------------------------------------------------------------------------------------------------------
;kidney compartment
;AK =  amount of STX in kidney tissue, umol
AK' = QK*(CB-CVK) -AGF'
Init AK = 0
CK = AK/VK
CVK = CK/PK

;AGF = amount of STX cleared in urine via glomerular filtration, umol
AGF' = CVK*GF
Init AGF = 0
;-------------------------------------------------------------------------------------------------------------------
;fat compartment
;AF = amount of STX in fat tissue, umol
AF' = QF*(CB-CVF)
Init AF = 0
CF = AF/VF
CVF = CF/PF
;-------------------------------------------------------------------------------------------------------------------
;tissue compartment (rapidly perfused tissues)
;AR = amount of STX in rapidly perfused tissues, umol
AR' = QR*(CB-CVR)
Init AR = 0
CR = AR/VR
CVR = CR/PR
;-------------------------------------------------------------------------------------------------------------------
;tissue compartment (slowly perfused tissues)
;AS = amount of STX in slowly perfused tissues, umol
AS' = QS*(CB-CVS)
Init AS = 0
CS = AS/VS
CVS = CS/PS
;-------------------------------------------------------------------------------------------------------------------
;blood compartment
;AB = amount of STX in blood, umol
AB' = QF*CVF +  QL*CVL  + QK*CVK + QS*CVS + QR*CVR + kn*ANe - (QF+QL+QK+QS+QR)*CB
Init AB = 0
CB = AB/VB                                   ; (umol/L), blood STX concentration
```

```
;======================================================================
;Mass balance calculations
;======================================================================
Total = IVDOSEumol + ODOSEumol * Fa
Calculated =  ANe + ASt + AL + AF + AK + AS + AR  + AB  + AGF

ERROR=((Total-Calculated)/Total+1E-30)*100
MASSBBAL=Total-Calculated + 1
```

Human model

```
;Date: Dec-9-2022
;Purpose: PBK model Saxitoxin (STX), built with in vitro and in silico derived parameters
;Species: Human
;Compiled by: Jiaqi Chen and Annelies Noorlander
;Organization: Wageningen University

;======================================================================
;Physiological parameters
;======================================================================
;tissue volumes (Brown et al. 1997)
BW = 60                     ; (kg), body weight used by EFSA (2009) and FAO/IOC/WHO (2004) when setting ARfD values
VFc = 0.214                 ; fraction of fat tissue
VLc = 0.026                 ; fraction of liver tissue
VKc = 0.004                 ; fraction of kidney tissue
VBc = 0.079                 ; fraction of blood
VRc = 0.064                 ; fraction of rapidly perfused tissues
VSc = 0.613                 ; fraction of slowly perfused tissues
                            ; total fraction is 1
VF = VFc*BW                 ; (L or kg), volume of fat tissue (calculated)
VL = VLc*BW                 ; (L or kg), volume of liver tissue (calculated)
VK = VKc*BW                 ; (L or kg), volume of kidney tissue (calculated)
VB = VBc*BW                 ; (L or kg), volume of blood (calculated)
VR = VRc*BW                 ; (L or kg), volume of rapidly perfused tissues (calculated)
VS = VSc*BW                 ; (L or kg), volume of slowly perfused tissues (calculated)
;----------------------------------------------------------------------------------------------------------------------
;blood flow rates (Brown et al. 1997)
QC = 15*BW^0.74             ; (L/hr), cardiac output
QFc = 0.052                 ; fraction of blood flow to fat
QLc = 0.227                 ; fraction of blood flow to liver
QKc = 0.175                 ; fraction of blood flow to kidney
QRc = 0.255                 ; fraction of blood flow to rapidly perfused tissues
QSc = 0.291                 ; fraction of blood flow to slowly perfused tissues
                            ; total fraction is 1
QF = QFc*QC                 ; (L/hr), blood flow to fat tissue (calculated)
QL = QLc*QC                 ; (L/hr), blood flow to liver tissue (calculated)
QK = QKc*QC                 ; (L/hr), blood flow to kidney tissue (calculated)
QR = QRc*QC                 ; (L/hr), blood flow to rapidly perfused tissues (calculated)
QS = QSc*QC                 ; (L/hr), blood flow to slowly perfused tissues (calculated)

;======================================================================
;Partition Coefficients
;======================================================================
;tissue/blood partition coefficients were obtained by dividing tissue/plasma partition coefficients (Rodgers and Rowland method, QIVIVE tool, version 2.0, Punt et al. 2021) by corresponding blood/plasma ratio (BPr), which is assumed to be 1 for basic compounds (Cubitt et al. 2009)
;LogP  = -4.6               ; WHO (2020)
;pKa: 8.22, 11.28           ; Rogers and Rapoport. 1980
PF = 0.17                   ; fat/blood partition coefficient
PL = 2.88                   ; liver/blood partition coefficient
PK = 2.51                   ; kidney/blood partition coefficient
PR = 2.80                   ; rapidly perfused tissues/blood partition coefficient
PS = 3.04                   ; slowly perfused tissues/blood partition coefficient

;======================================================================
;Kinetic parameters
;======================================================================
;Fa=Fraction absorbed
Fa = 0.36                   ; calculated with QSAR-based equations
;Absorption constant
ka  = 0.14                  ; (/hr), calculated with QSAR-based equations
;----------------------------------------------------------------------------------------------------------------------
;Excretion from kidney via glomerular filtration
;Glomerular filtration
GFR = 1.8                                          ; (mL/min/kg bw), Walton et al., 2004
GF = GFR/1000*BW*60                                ; (L/hr), human glomerular filtration rate

;======================================================================
;Run settings
;======================================================================
;Molecular weight
MW = 299.29                                        ; (g/mol), molecular weight STX (PubChem)

;Oral dose
ODOSEmg = 0.0005                                   ; (mg/kg bw), given oral dose in mg/kg bw
```

```
ODOSEumol2 = ODOSEmg*1E-3/MW*1E6                ; (umol/kg bw), given oral dose recalculated to umol/kg bw
ODOSEumol=ODOSEumol2*BW                         ; (umol), given oral dose in umol

;time
Starttime = 0          ; in hr
Stoptime = 24          ; in hr

;====================================================================
;Model calculations
;====================================================================
;STX model
;--------------------------------------------------------------------
;GI-tract compartment
;ASt  = amount of STX in GI-tract, umol
Ast'  = -ka*Ast
Init ASt =ODOSEumol*Fa
;--------------------------------------------------------------------
;liver compartment
;AL = amount of STX in liver tissue, umol
AL' = ka*ASt + QL*(CB - CVL)
Init AL = 0
CL = AL/VL
CVL = CL/PL
;--------------------------------------------------------------------
;kidney compartment
;AK =  amount of STX in kidney tissue, umol
AK' = QK*(CB-CVK) -AGF'
Init AK = 0
CK = AK/VK
CVK = CK/PK

;AGF = amount of STX cleared in urine via glomerular filtration, umol
AGF' = CVK*GF
Init AGF = 0
;--------------------------------------------------------------------
;fat compartment
;AF = amount of STX in fat tissue, umol
AF' = QF*(CB-CVF)
Init AF = 0
CF = AF/VF
CVF = CF/PF
;--------------------------------------------------------------------
;tissue compartment (rapidly perfused tissues)
;AR = amount of STX in rapidly perfused tissues, umol
AR' = QR*(CB-CVR)
Init AR = 0
CR = AR/VR
CVR = CR/PR
;--------------------------------------------------------------------
;tissue compartment (slowly perfused tissues)
;AS = amount of STX in slowly perfused tissues, umol
AS' = QS*(CB-CVS)
Init AS = 0
CS = AS/VS
CVS = CS/PS
;--------------------------------------------------------------------
;blood compartment
;AB = amount of STX in blood, umol
AB' = QF*CVF +QL*CVL  +QK*CVK +QS*CVS +QR*CVR -(QF+QL+QK+QS+QR)*CB
Init AB = 0
CB = AB/VB                                      ; (umol/L), blood STX concentration

;====================================================================
;Mass balance calculations
;====================================================================
Total = ODOSEumol * Fa
Calculated =  ASt + AL + AF + AK + AS + AR  + AB  + AGF

ERROR=((Total-Calculated)/Total+1E-30)*100
MASSBBAL=Total-Calculated + 1
```

References

Aballay-González, A.; Gallardo-Rodriguez, J.J.; Silva-Higuera, M.; Rivera, A.; Ulloa V.; Delgado-Rivera, L.; Rivera-Belmar, A.; Astuya, A.; Neuro-2a cell-based assay for toxicity equivalency factor - proposal and evaluation in Chilean contaminated shellfish samples. *Food Addit. Contam. Part A*. **2020**, 37(1), 162-173.

Alonso, E.; Alfonso, A.; Vieytes, M.R.; Botana, L.M. Evaluation of toxicity equivalent factors of paralytic shellfish poisoning toxins in seven human sodium channels types by an automated high throughput electrophysiology system. *Arch. Toxicol*. **2016**, 90, 479-488.

Andresen B.M.; Bois, J.D. *De novo* synthesis of modified saxitoxins for sodium ion channel study. *J. Am. Chem. Soc*. **2009**, 131(35), 12524-12525.

Brown, R.P.; Delp, M.D.; Lindstedt, S.L.; Rhomberg, L.R.; Beliles, R.P. Physiological parameter values for physiologically based pharmacokinetic models. *Toxicol. Ind. Health*. **1997**, 13(4), 407-484.

Cañete, E.; Diogène, J. Comparative study of the use of neuroblastoma cells (Neuro-2a) and neuroblastoma × glioma hybrid cells (NG108-15) for the toxic effect quantification of marine toxins. *Toxicon*. **2008**, 52, 541-550.

Cubitt, H.E.; Houston, J.B.; Galetin, A. Relative importance of intestinal and hepatic glucuronidation—Impact on the prediction of drug clearance. *Pharm Res*. **2009**, 26, 1073-1083.

Doucette, G.J.; Logan, M.M.; Ramsdell, J.S.; Van Dolah, F.M. Development and preliminary validation of a microtiter plate-based receptor binding assay for paralytic shellfish poisoning toxins. *Toxicon*, **1997**, 35(5), 625-36.

EFSA. Scientific opinion of the panel on contaminants in the food chain on a request from the European Commission on marine biotoxins in shellfish – Saxitoxin group. *EFSA J*. **2009**, 1019, 1-76.

FAO/IOC/WHO. Report of the Joint FAO/IOC/WHO Ad Hoc Expert Consultation on biotoxins in bivalve molluscs. Oslo, Norway. **2004**.

Finch, S.C.; Boundy, M.J.; Harwood, D.T. The acute toxicity of tetrodotoxin and tetrodotoxin–saxitoxin mixtures to mice by various routes of administration. *Toxins*. **2018**, 10(11), 423.

Hall, C.; Lueshen, E.; Mošat', A.; Linninger, A.A. Interspecies scaling in pharmacokinetics: a novel whole-body physiologically based modeling framework to discover drug biodistribution mechanisms *in vivo*. *J. Pharm. Sci*. **2012**, 101(3), 1221-1241.

Hoehne, A.; Behera, D.; Parsons, W.H.; James, M.L.; Shen, B.; Borgohain, P.; Bodapati, D.; Prabhakar, A.; Gambhir, S.S.; Yeomans, D.C.; Biswal, S.; Chin, F.T.; Bois, J.D. A ^{18}F-Labeled saxitoxin derivative for *in vivo* PET-MR imaging of voltage-gated sodium channel expression following nerve injury. *J. Am. Chem. Soc*. **2013**, 135, 18012-18015.

Kulagina, N.V.; O'Shaughnessy, T.J.; Ma, W.; Ramsdell, J.S.; Pancrazio, J.J. Pharmacological effects of the marine toxins, brevetoxin and saxitoxin, on murine frontal cortex neuronal networks. *Toxicon*. **2004**, 44, 669-676.

Llewellyn, L.; Negri, A.; Quilliam, M. High affinity for the rat brain sodium channel of newly discovered hydroxybenzoate saxitoxin analogues from the dinoflagellate *Gymnodinium catenatum*. *Toxicon*. **2004**, 43, 101-104.

Melegari, S.P.; Pinto, C.R.S.C.; Moukha, S.; Creppy,E.E.; Matias, W.G. Evaluation of cytotoxicity and cell death induced *in vitro* by saxitoxin in mammalian cells. *J. Toxicol. Environ. Health A*. **2015**, 78(19), 1189-1200.

Moran, O.; Picollo, A.; Conti. F.; Tonic and phasic guanidinium toxin-block of skeletal muscle Na channels expressed in mammalian cells. *Biophy. J*. **2003**, 84, 2999-3006.

Munday, R.; Thomas, K.; Gibbs, R.; Murphy, C.; Quilliam, M.A. Acute toxicities of saxitoxin, neosaxitoxin, decarbamoyl saxitoxin and gonyautoxins 1&4 and 2&3 to mice by various routes of administration. *Toxicon*. **2013**, 76(15), 77-83.

Nicholson, R.A.; Li, G.H.; Buenaventura, E.; Graham, D. A rapid and sensitive assay for paralytic shellfish poison (PSP) toxins using mouse brain synaptoneurosomes. *Toxicon*. **2002**, 40(6), 831-838.

Nicolas, J.; Hendriksen, P.J.M.; van Kleef, R.G.D.M.; de Groot, A.; Bovee, T.F.H.; Rietjens, I.M.C.M.; Westerink, R.H.S. Detection of marine neurotoxins in food safety testing using a multielectrode array. *Mol. Nutr. Food Res*. **2014**, 58, 2369-2378.

Nguyen, H.V.N.; Smith M.E.; Swoboda H.D. Shellfish toxicity (Last update: July 18, 2022). https://www.ncbi.nlm.nih.gov/books/NBK470225/ (Accessed 8 August 2022).

Ondrus, A.E.; Lee, H.D.; Iwanaga, S.; Parsons, W.H.; Andresen, B.M.; Moerner, W.E.; Bois, J.D. Fluorescent saxitoxins for live cell imaging of single voltage-gated sodium ion channels beyond the optical diffraction limit. *Chem Biol*. **2012**, 19(7), 902-912.

Perez, S.; Vale, C.; Botana, A.M.; Alonso, E.; Vieytes, M.R.; Botana, L.M. Determination of toxicity equivalent factors for paralytic shellfish toxins by electrophysiological measurements in cultured neurons. *Chem. Res. Toxicol*. **2011**, 24, 1153-1157.

Perreault, F.; Matias, M.S.; Melegari, S.P.; Pinto, C.R.S.C.; Creppy, E.E.; Popovic, R.; Matias, W.G. Investigation of animal and algal bioassays for reliable saxitoxin ecotoxicity and cytotoxicity risk evaluation. *Ecotoxicol. Environ. Saf*. **2011**, 74, 1021-1026.

Punt, A.; Pinckaers, N.; Peijnenburg, A.; Louisse, J. Development of a web-based toolbox to support quantitative *in-vitro*-to-*in-vivo* extrapolations (QIVIVE) within nonanimal testing strategies. *Chem. Res. Toxicol*. **2021**, 34, 460-472.

Rogers, R.S.; Rapoport, H. The pKa's of saxitoxin. *J. Am. Chem. Soc*. **1980**, 102, 7335-7339.

Stafford, R.G.; Hines, H.B. Urinary elimination of saxitoxin after intravenous injection. *Toxicon*. **1995**, 33(11), 1501-1510.

Suarez-Isla, B.A. Saxitoxin and other paralytic toxins: Toxicological profile. In *Marine and Freshwater Toxins*; Gopalakrishnakone, P., Haddad Jr., V., Tubaro, A., Kim, E., Kem, W. Eds.; Springer: Dordrecht, the Netherlands, **2016**; pp 23-41.

Thermo Fisher. Media supplement guide (rev 1-Mar-2011). https://www.thermofisher.com/nl/en/home/references/protocols/drug-discovery/adme-tox-protocols/media-supplement-guide.html#1. (Accessed 8 August 2022).

Usup, G.; Leaw, C.-P.; Cheah, M.-Y.; Ahmad, A.; Ng, B.-K. Analysis of paralytic shellfish poisoning toxin congeners by a sodium channel receptor binding assay. *Toxicon*. **2004**, 44, 37-43.

Vale, C.; Alfonso, A.; Vieytes, M.R.; Romarís, X.M.; Arévalo, F.; Botana, A.M.; Botana, L.M. *In vitro* and *in vivo* evaluation of paralytic shellfish poisoning toxin potency and the influence of the pH of extraction. *Anal. Chem*. **2008**, 80, 1770-1776.

Vélez, P.; Sierralta, J.; Alcayaga, C.; Fonseca, M.; Loyola, H.; Johns, D.C.; Tomaselli, G.F.; Marbán, E.; Suárez-Isla, B.A. A functional assay for paralytic shellfish toxins that uses recombinant sodium channels. *Toxicon*. **2001**, 39, 929-935.

Viallon, J.; Chinain M.; Darius, H.T. Revisiting the neuroblastoma cell-based sssay (CBA-N2a) for the improved detection of marine toxins active on voltage gated sodium channels (VGSCs). *Toxins*. **2020**, 12, 281.

Walker, J.R.; Novick, P.A.; Parsons, W.H.; McGregor, M.; Zablocki, J.; Pande, V.S.; Bois, J.D. Marked difference in saxitoxin and tetrodotoxin affinity for the human nociceptive voltage-gated sodium channel (Nav1.7). *PNAS*, **2012**, 109(52), 21551.

Walton, K.; Dorne, J.L.C.M.; Renwick. A.G. Species-specific uncertainty factors for compounds eliminated principally by renal excretion in humans. *Food and Chem. Toxicol*, **2004**, 42, 261-274.

WHO. Cyanobacterial toxins: saxitoxins. Background document for development of WHO guidelines for drinking-water quality and guidelines for safe recreational water environments. Geneva, Switzerland. **2020**.

WHO. Characterization and application of physiologically based pharmacokinetic models in risk assessment. Geneva, Switzerland. **2010**.

Yamagata, T.; Zanelli, U.; Gallemann, D.; Perrin, D.; Dolgos, H; Petersson, C. Comparison of methods for the prediction of human clearance from hepatocyte intrinsic clearance for a set of reference compounds and an external evaluation set. *Xenobiotica*. **2017**, 47(9), 741-751.
